# Finding prognostic gene pairs for cancer from patient-specific gene networks

**DOI:** 10.1186/s12920-019-0634-0

**Published:** 2019-12-20

**Authors:** Byungkyu Park, Wook Lee, Inhee Park, Kyungsook Han

**Affiliations:** Department of Computer Engineering, Inha University, Incheon, 22212 South Korea

**Keywords:** Patient-specific gene network, Prognostic gene pair, Cancer, Dynamic visualization

## Abstract

**Background:**

Molecular characterization of individual cancer patients is important because cancer is a complex and heterogeneous disease with many possible genetic and environmental causes. Many studies have been conducted to identify diagnostic or prognostic gene signatures for cancer from gene expression profiles. However, some gene signatures may fail to serve as diagnostic or prognostic biomarkers and gene signatures may not be found in gene expression profiles.

**Methods:**

In this study, we developed a general method for constructing patient-specific gene correlation networks and for identifying prognostic gene pairs from the networks. A patient-specific gene correlation network was constructed by comparing a reference gene correlation network from normal samples to a network perturbed by a single patient sample. The main difference of our method from previous ones includes (1) it is focused on finding prognostic gene pairs rather than prognostic genes and (2) it can identify prognostic gene pairs from gene expression profiles even when no significant prognostic genes exist.

**Results:**

Evaluation of our method with extensive data sets of three cancer types (breast invasive carcinoma, colon adenocarcinoma, and lung adenocarcinoma) showed that our approach is general and that gene pairs can serve as more reliable prognostic signatures for cancer than genes.

**Conclusions:**

Our study revealed that prognosis of individual cancer patients is associated with the existence of prognostic gene pairs in the patient-specific network and the size of a subnetwork of the prognostic gene pairs in the patient-specific network. Although preliminary, our approach will be useful for finding gene pairs to predict survival time of patients and to tailor treatments to individual characteristics. The program for dynamically constructing patient-specific gene networks and for finding prognostic gene pairs is available at http://bclab.inha.ac.kr/pancancer.

## Background

Cancer is a very heterogeneous and complex disease with many possible genetic and environmental causes. The same treatment for the same type of cancer often results in different outcomes in terms of efficacy and side effects of the treatment. Many targeted therapies are effective only for patients with specific genetic alterations (known as driver mutations) that help cancer cells form and grow [1, 2]. Therefore, identifying genetic mutations specific to individual cancer patients is important for determining targeted therapies that can effectively cure the patients while minimizing side effects [3].

For the past decade, a huge amount of gene expression data have been generated by high-throughput technologies such as microarray and RNA-seq. The availability of the data has triggered the development of a variety of computational methods for cancer research. For example, several methods have been developed for exploring gene expression characteristics [4–8] or for constructing gene networks of several types (e.g., gene co-expression network, gene correlation network, or gene regulatory network). However, a patient-specific gene correlation network is not easy to construct from a single sample because a gene network requires many samples to compute gene-gene relations. Recently Liu et al. [9] and a few others proposed a method to construct a sample-specific network by computing the difference between a reference network for multiple reference samples and a network perturbed by a new sample. But, their sample-specific network is not reliable because a slight change to the reference samples can result in a significantly different sample-specific network for the same sample due to the small number of reference samples.

In this study, we developed a new method for constructing cancer patient-specific gene correlation networks and for finding potential prognostic gene pairs. So far many computational methods developed for cancer research have focused on identifying diagnostic or prognostic gene signatures from gene expression data which can serve as diagnostic or prognostic biomarkers. However, such gene signatures may not be found in gene expression data because gene expression levels are often sensitive to systematic biases of measurements [10]. One objective of our method is to find prognostic gene pairs which can be used to predict the likely outcome or survival time of cancer patients. It should be noted that the network built by our method is not a gene regulatory network because our network does not show regulatory relations between genes. It is also different from a typical gene co-expression network that represents co-expression relations between genes.

As shown later in this paper, our approach has been used in constructing patient-specific gene networks for three types of cancer (breast invasive carcinoma, colon adenocarcinoma, and lung adenocarcinoma). From the gene-gene relations computed for the networks, we identified significant gene pairs in each cancer type. The results of evaluating our method demonstrated that informative patient-specific networks can be constructed dynamically from the user’s choice of a sample and that significant prognostic gene pairs can be found even when no significant prognostic genes exist. The remainder of this paper presents details of our method and experimental results of the method in three types of cancer.

## Methods

This section discusses our approach to constructing cancer patient-specific networks and identifying prognostic gene pairs of cancer patients.

### Data sets

For comparative analysis of cancer samples and normal samples, we obtained gene expressions of tumor samples from The Cancer Genome Atlas (TCGA) [11] and gene expressions of normal samples from Genotype-Tissue Expression (GTEx) [12]. For tumor samples, we selected primary tumor samples of three types: breast invasive carcinoma (BRCA), colon adenocarcinoma (COAD), and lung adenocarcinoma (LUAD). For normal samples related to each cancer type, we excluded cell lines and selected normal tissues in breast, colon, and lung from the GTEx dataset. All the gene expression data of the samples were extracted from the UCSC TOIL RNA-seq recompute compendium (https://toil.xenahubs.net) [13]. The gene expressions were processed using RSEM [14] and log2-transformed.

For each type of cancer, we collected elite genes and related genes from the MalaCards database [15]. We obtained a total of 516 genes associated with BRCA (74 elite and 442 related genes), 466 genes for COAD (70 elite and 396 related genes), and 410 genes for LUAD (61 elite and 349 related genes). Table 1 shows the number of samples used in our study, and the list of the genes is available in Additional file 1.

**Table 1:** The number of samples and genes used in this study

Cancer type	ID	Number of samples		Number of
		Tumor	Normal	Malacards genes
Breast invasive carcinoma	BRCA	1050	100	516
Colon adenocarcinoma	COAD	286	100	466
Lung adenocarcinoma	LUAD	405	100	410

### Constructing cancer patient-specific gene networks

For every pair of genes in normal samples we computed the Pearson correlation coefficient (PCC) between their expression levels by equation 1. In the equation, *N* is the number of samples and $\bar {x}$ is the mean of *x*. A reference gene network for *N* normal samples was constructed for each type of cancer.

1 $$  PCC(x_{i}, x_{j}) = \frac{\sum_{k=1}^{N}{(x_{ik}-\bar{x}_{i})(x_{jk}-\bar{x}_{j})}}{\sqrt{\sum_{k=1}^{N}{(x_{ik} -\bar{x}_{i})^{2}}\sum_{k=1}^{N}{(x_{jk}-\bar{x}_{j})^{2}}}}  $$

**Fig. 1:**
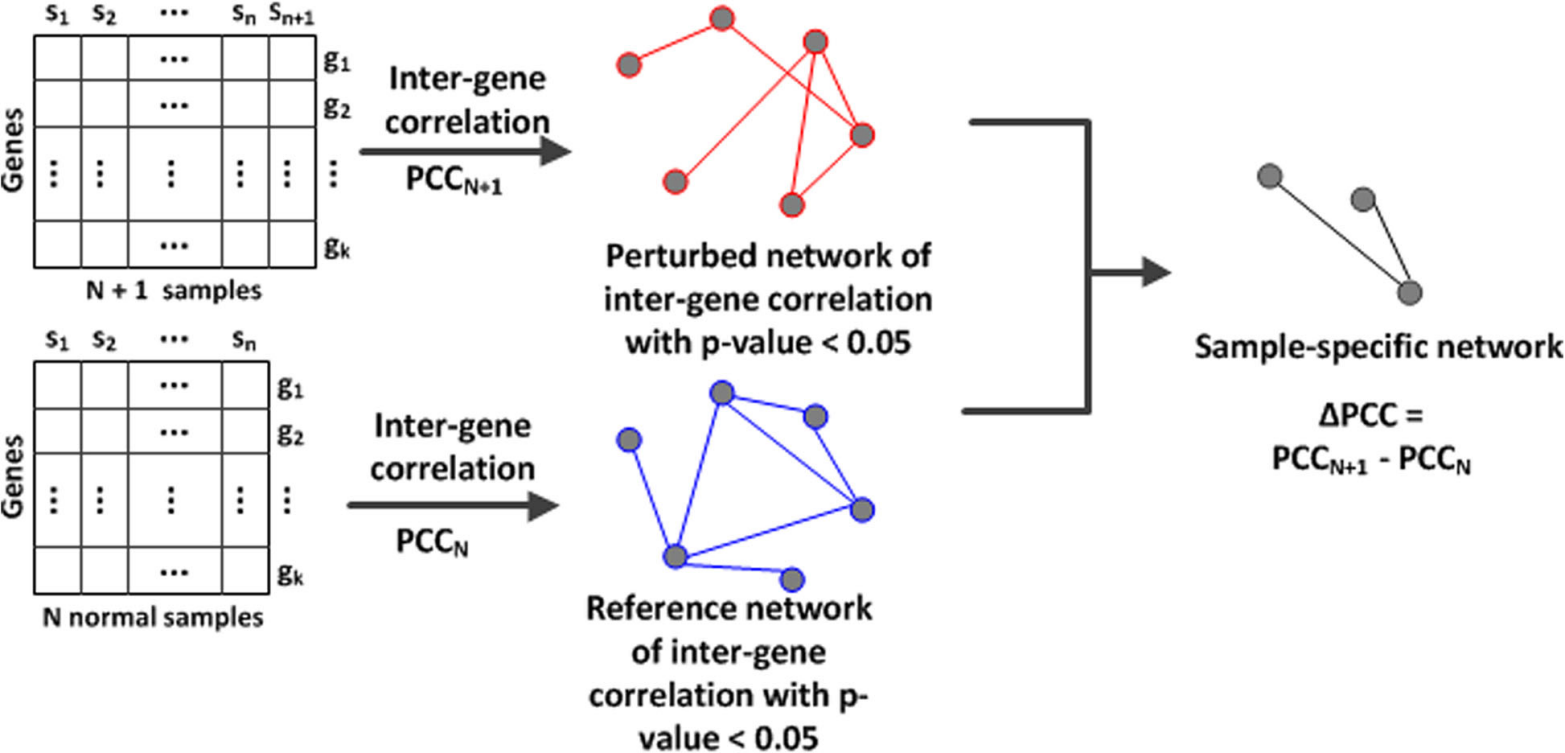
Process of constructing cancer patient-specific gene network. A reference gene network is first constructed from *N* normal samples. A perturbed network is obtained by adding a cancer sample of the patient to the *N* normal samples. A patient-specific gene network is obtained by subtracting the reference network from the perturbed network

For a patient-specific gene network, we first constructed a perturbed network by adding a single sample of the patient to the *N* normal samples. A patient-specific gene correlation network was obtained by subtracting the reference network from the perturbed network (Fig. 1). For every pair of genes *g*_*i*_ and *g*_*j*_, we computed the change in PCC between the perturbed network and reference network by equation 2. In the patient-specific network, *Δ*PCC reflects the difference in gene correlations between the normal samples and the patient sample.

2 $$  {\Delta PCC(g_{i}, g_{j}) = |PCC_{perturbed}(g_{i}, g_{j}) - PCC_{reference}(g_{i}, g_{j})|}  $$

### Finding prognostic gene pairs for cancer patients

To find potential prognostic gene pairs, we clustered the patients into two groups by hierarchical clustering in the following way. For each gene pair in a sample of a cancer patient, we examined whether *Δ*PCC of the gene pair belongs to the top 25% of the corresponding pair in all tumor samples. Patients with the top 25% *Δ*PCC of a gene pair were clustered into one group, and the remaining patients were clustered into the other group. When either one of the groups contains <10 patients, the gene pair was excluded from potential prognostic gene pairs.

We performed the log-rank test [16] using the lifelines package (https://lifelines.readthedocs.io). For every gene pair, we examined the difference in their survival time and obtained the *p*-value of the test. It should be noted that patients can be clustered differently depending on the gene pair used for clustering. The *p*-value of the log-rank test was adjusted using the Benjamini-Hochberg procedure [17] in a Python package (https://www.statsmodels.org), which consists of the following steps to control the false discovery rate (FDR) at level *α*. In the second step, $p_{j} \leq \frac {j}{m}\alpha $ can be transformed to $p_{j} \frac {m}{j} \leq \alpha $, so $min(1, p_{j}\frac {m}{j})$ was used as an adjusted *p*-value of the log-rank test.

1. Sort the *p*-values as *p*_1_,*p*_2_,..., *p*_*m*_.
2. Find the rank *j* for which $p_{j} \leq \frac {j}{m}\alpha $.
3. Declare the top *j* tests 1, 2,..., j as significant.

As for criteria for selecting potential prognostic gene pairs, we used the *p*-value of the log-rank test and correlations between two genes in total tumor samples. Only the gene pairs with an adjusted *p*-value of the log-rank test <0.05 and *p*-value of PCC <0.05 were selected as potential prognostic gene pairs.

## Results

### Patient-specific gene networks

For *k* genes in *n* tumor samples, we computed $n {k \choose 2} \Delta $PCCs. We computed $1050\cdot {516 \choose 2}=139,513,500 \Delta $PCCs for breast cancer, $286\cdot {466 \choose 2}=30,986,670 \Delta $PCCs for colon cancer, and $405\cdot {410 \choose 2}=33,957,225 \Delta $PCCs for lung cancer (see Table 1 for the number of tumor samples and genes). Among the *Δ*PCCs, gene pairs with the *p*-value of PCC <0.05 in both the reference network and the perturbed network were selected. There were a total of 44,275 distinct gene pairs for breast cancer, 43,577 distinct gene pairs for colon cancer, and 16,874 distinct gene pairs for lung cancer. The gene pairs are available in Additional file 2.

For dynamic visualization of patient-specific gene networks, we built a graph database with the gene pairs and their *Δ*PCC values. We developed a web-based system using javascript (https://github.com/neo4j-contrib/neovis.js), which dynamically visualizes cancer patient-specific gene networks. Users of the web-based system can search gene pairs either in group 1 or group 2. As discussed earlier, group 1 is a set of samples with a gene pair that show a relatively large change in PCC from normal samples and group 2 is a set of the remaining samples. The system is available at http://bclab.inha.ac.kr/pancancer.

### Prognostic gene pairs in breast cancer

**Fig. 2:**
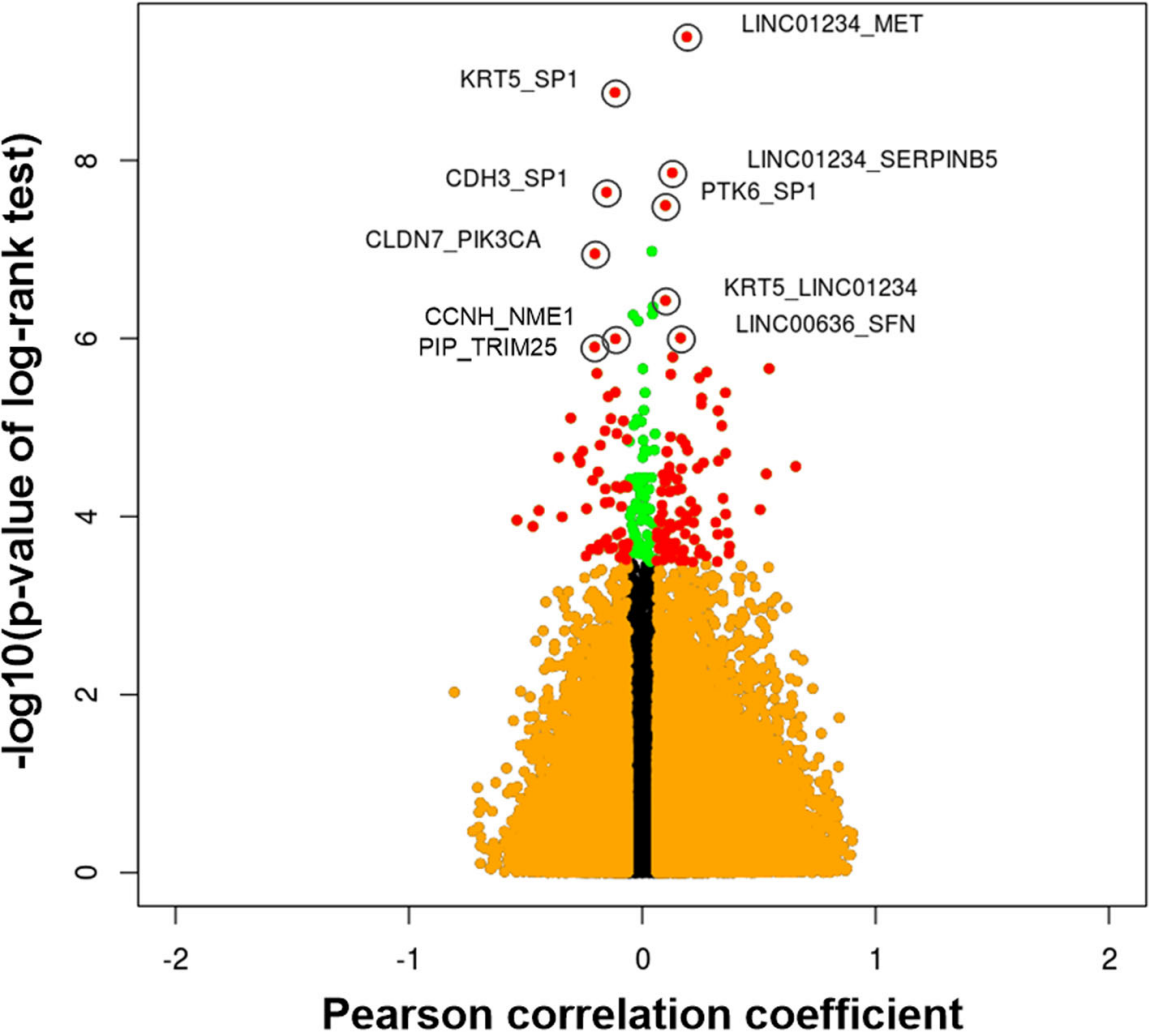
Scatter plot of the *p*-value of the log-rank test against the Pearson correlation coefficient (PCC) of gene pairs in breast cancer. Only the gene pairs with an adjusted *p*-value of the log-rank test <0.05 and *p*-value of PCC <0.05 were selected as potential prognostic gene pairs (red dots)

**Fig. 3:**
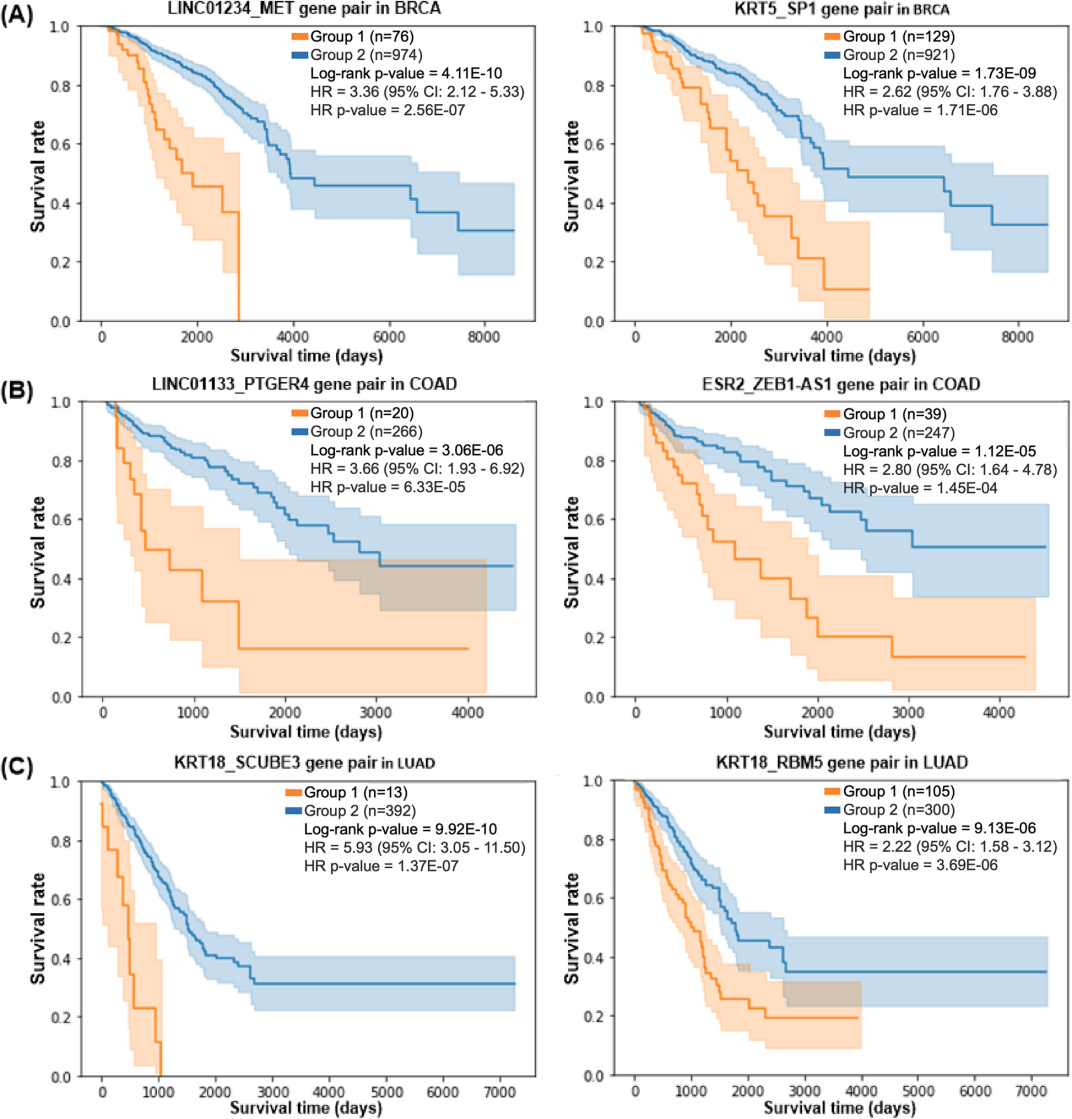
Kaplan-Meier plots comparing the survival rates of two groups with respect to prognostic gene pairs. **a** The survival rates of two groups of BRCA samples with respect to prognostic gene pairs LINC01234_MET (left plot) and KRT5_SP1 (right plot). **b** The survival rates of two groups of COAD samples with respect to prognostic gene pairs LINC01133_PTGER4 (left plot) and ESR2_ZEB1-AS1 (right plot). **c** The survival rates of two groups of LUAD samples with respect to prognostic gene pairs KRT18_SCUBE3 (left plot) and KRT18_RBM5 (right plot). In all Kaplan-Meier plots, group 1 is a set of cancer samples with a big change in PCC of the corresponding gene pair from the normal samples, and group 2 is a set of the remaining samples of the same cancer type. Group 1 consistently shows a lower survival rate than group 2

There were a total of 44,275 gene pairs in breast cancer samples (Additional file 2). We ranked the gene pairs by the adjusted *p*-value of the log-rank test, and then selected those with an adjusted *p*-value of the log-rank test <0.05 and *p*-value of PCC <0.05 as potential prognostic gene pairs in breast cancer (Fig. 2). The selected gene pairs are listed in Additional file 3 and top 10 gene pairs are shown in Table 2. For the top two gene pairs in Table 2 (LINC01234_MET and KRT5_SP1), Fig. 3a shows the survival rate of two groups using Kaplan-Meier plots [18].

**Table 2:** Gene pairs with the lowest adjusted *p*-value in breast cancer. Group 1: samples with the top 25% *Δ*PCC for the gene pair. Group 2: remaining samples

Gene pair	Group 1	Group 2	Log-rank test			Cox PH		
			statistic	*p*-value	adj. *p*-value	Hazard ratio	*p*-value	95% CI
LINC01234_MET	76	974	39.06	4.11E-10	7.98E-07	3.36	2.56E-07	2.12 - 5.33
KRT5_SP1	129	921	36.26	1.73E-09	2.92E-06	2.62	1.71E-06	1.76 - 3.88
LINC01234_SERPINB5	41	1009	32.21	1.38E-08	1.95E-05	3.82	4.40E-06	2.15 - 6.79
CDH3_SP1	25	1025	31.24	2.28E-08	2.95E-05	7.94	3.70E-08	3.79 - 16.61
PTK6_SP1	55	995	30.58	3.20E-08	3.83E-05	4.55	1.21E-06	2.46 - 8.39
CLDN7_PIK3CA	53	997	28.16	1.11E-07	1.23E-04	4.00	2.74E-07	2.36 - 6.80
KRT5_LINC01234	76	974	25.81	3.75E-07	3.89E-04	3.06	3.04E-06	1.91 - 4.91
LINC00636_SFN	13	1037	23.95	9.89E-07	8.69E-04	6.98	2.33E-05	2.83 - 17.20
CCNH_NME1	83	967	23.90	1.01E-06	8.75E-04	2.62	1.95E-05	1.68 - 4.09
PIP_TRIM25	41	1009	23.50	1.25E-06	1.04E-03	2.86	4.01E-04	1.59 - 5.13

Group 1 of breast cancer samples with a big change in PCC of LINC01234_MET (i.e., top 25% *Δ*PCC for LINC01234_MET) from the normal samples showed a much lower survival rate than the other group (group 2) of breast cancer samples (the left Kaplan-Meier plot in Fig. 3a). Likewise, group 1 of breast cancer samples with a big change in PCC of KRT5_SP1 from the normal samples revealed a lower survival rate than the other group of breast cancer samples (the right Kaplan-Meier plot in Fig. 3a).

For colon cancer, LINC01133_PTGER4 and ESR2_ZEB1-AS1 were found as potential prognostic gene pairs (Fig. 3b). In a similar way, KRT18_SCUBE3 and KRT18_RBM5 were found as potential prognostic gene pairs for lung cancer (Fig. 3c).

**Fig. 4:**
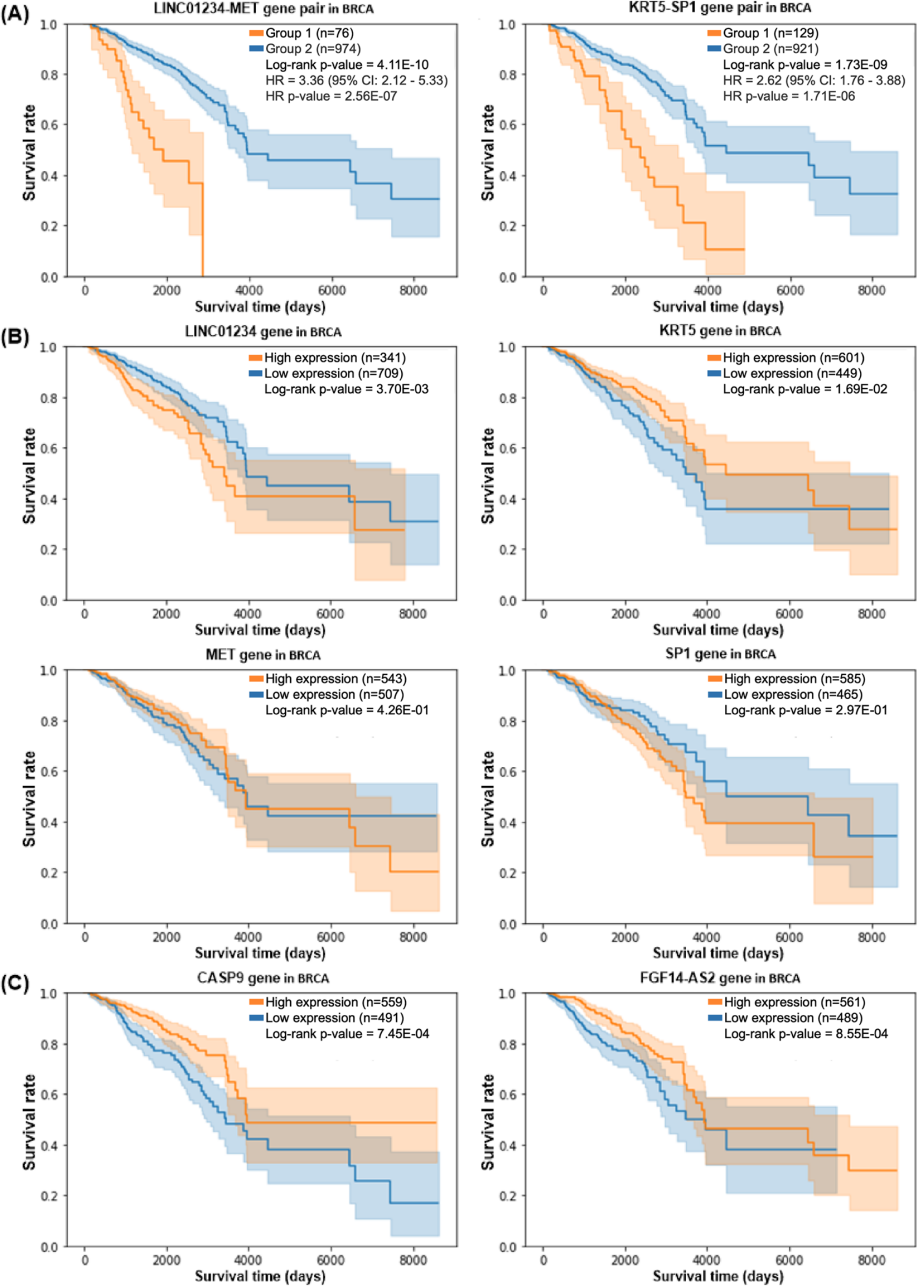
Comparison of the prognostic power of gene pairs and single genes in BRCA. **a** The survival rates of two groups of BRCA samples with respect to prognostic gene pairs LINC01234_MET and KRT5_SP1. **b** The survival rates of two groups of BRCA samples with respect to four single genes (LINC01234, MET, KRT5 and SP1) involved in the gene pairs of **a**. **c** The survival rates of two groups of BRCA samples with respect to two genes (CASP9 and FGF14-AS2) which showed the lowest adjusted *p*-value in the log-rank test. None of the 6 single genes (LINC01234, MET, KRT5, SP1, CASP9 and FGF14-AS2) are predictive of survival rates. Group 1: cancer samples with the top 25% *Δ*PCC of the corresponding gene pair from the normal samples. Group 2: the remaining cancer samples of the same cancer type. High expression: cancer samples with higher expression levels than the average expression level. Low expression: remaining cancer samples

For comparative purposes, we performed survival analysis with all individual genes. Figure 4a is the same Kaplan-Meier plots shown in Fig. 3a, and Fig. 4b shows four Kaplan-Meier plots comparing the survival rates with respect to four individual genes. The four Kaplan-Meier plots in Fig. 4b compare the survival rates of two groups with respect to four individual genes, LINC01234, MET, KRT5, and SP1, which are involved in prognostic gene pairs LINC01234_MET and KRT5_SP1. It is interesting to note that the genes no longer show prognostic power when they are used alone. Figure 4c shows the Kaplan-Meier plots for two genes, CASP9 and FGF14-AS2, which showed the lowest adjusted *p*-value among all single genes in the log-rank test. Despite the low *p*-value, the two genes (CASP9 and FGF14-AS2) do not show prognostic power for breast cancer. The results of survival analysis in breast cancer indicate that gene pairs can be more powerful prognostic biomarkers than individual genes.

**Fig. 5:**
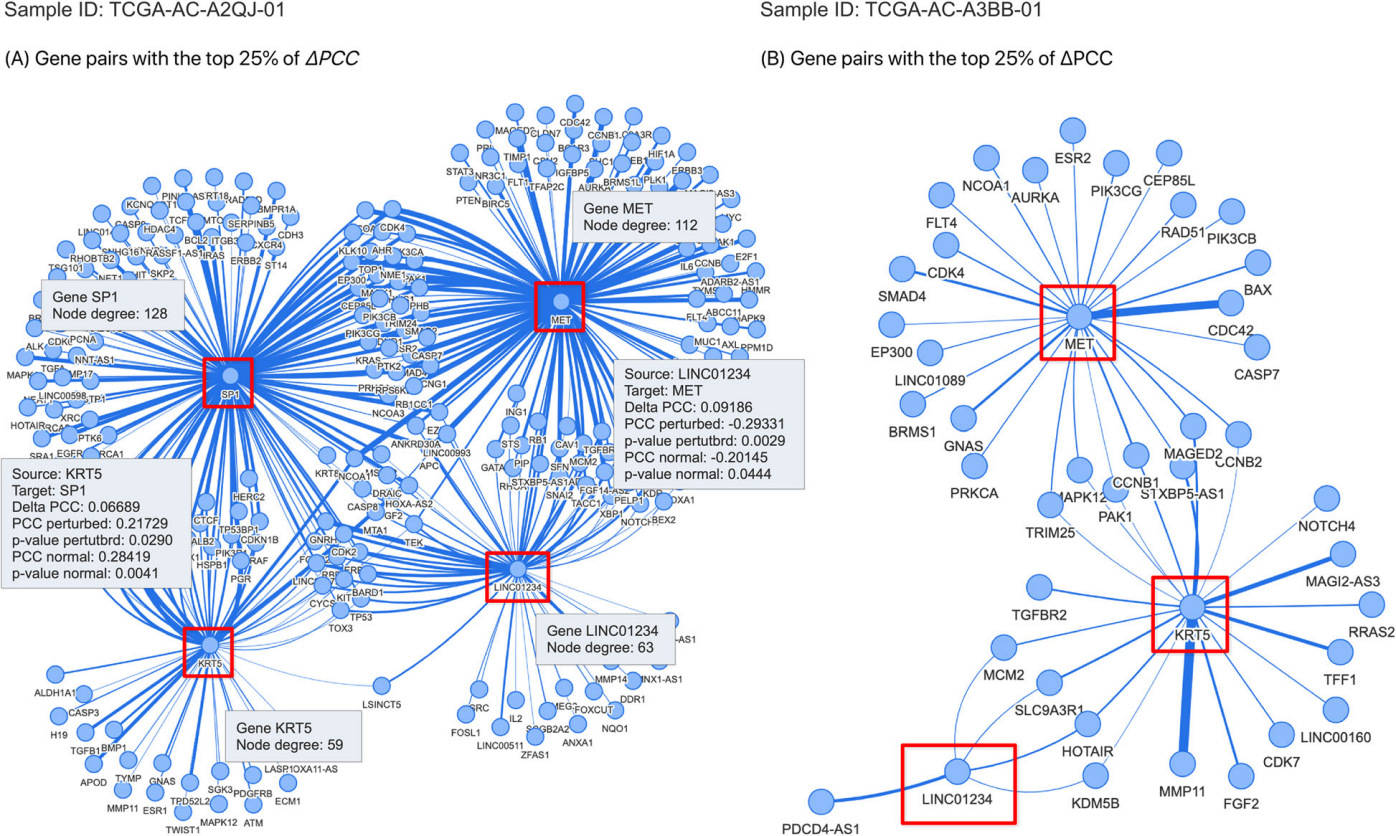
Example of a breast cancer patient-specific network. **a** Subnetwork of a patient-specific network for sample TCGA-AC-A2QJ-01 in group 1. **b** Subnetwork of a patient-specific network for sample TCGA-AC-A3BB-01 in group 2

The prognostic gene pairs for breast cancer can be included or excluded in a patient-specific gene network depending on the type of the patient. As an example, Figure 5 shows two subnetworks in the patient-specific gene networks for two breast cancer samples. Sample TCGA-AC-A2QJ-01 shows a big change in PCCs of two prognostic gene pairs LINC01234_MET and KRT5_SP1 (group 1 in the survival analysis) and the patient-specific network for the sample (Fig. 5A) includes the edges corresponding to the gene pairs. In contrast, sample TCGA-AC-A3BB-01 shows a much smaller change in PCCs of LINC01234_MET and KRT5_SP1 (group 2). In the patient-specific network for the sample TCGA-AC-A3BB-01 of group 2, both prognostic gene pairs LINC01234_MET and KRT5_SP1 are missing (Figure 5b). In addition to this, the subnetwork in Fig. 5b is much smaller than that in Figure 5a. The results imply that the survival rate of cancer patients is associated with the existence of prognostic gene pairs in the patient-specific network and the size of a subnetwork of prognostic gene pairs in the patient-specific network. This is because a larger subnetwork is likely to contain more prognostic gene pairs than a smaller subnetwork.

The LINC01234 gene is a long non-coding RNA (lncRNA) signature which is pervasive in different stages, subtypes and age groups of breast cancer [19]. In the log-rank test, gene LINC01234 alone attained the 11-th rank with respect to the adjusted *p*-value (Table 3) and was not a significant prognostic gene. However, a gene pair involving LINC01234 (i.e., LINC01234_MET) was the most significant prognostic gene pair for breast cancer.

**Table 3:** Genes with the lowest adjusted *p*-value in breast cancer. High expression: the number of samples with a higher expression level than the average log2-expression level. Low expression: remaining samples

Gene	High expression	Low expression	Log-rank statistic	*p*-value	adj. *p*-value
CASP9	559	491	11.37	0.0007	0.1049
FGF14-AS2	561	489	11.12	0.0009	0.1049
NTRK3	514	536	10.77	0.0010	0.1049
IL2	472	578	10.63	0.0011	0.1049
BRMS1L	506	544	10.34	0.0013	0.1049
KRT14	616	434	10.27	0.0014	0.1049
PTK2	474	576	10.04	0.0015	0.1049
GLI1	534	516	9.93	0.0016	0.1049
RBBP8	490	560	8.67	0.0032	0.1647
ADARB2-AS1	229	821	8.65	0.0033	0.1647
LINC01234	341	709	8.43	0.0037	0.1647
CLCA2	479	571	8.36	0.0038	0.1647
ST8SIA6-AS1	389	661	7.29	0.0069	0.2528

So far, no or little relation between LINC01234 and MET has been known, but our method found the gene pair LINC01234_MET as the most significant prognostic gene pair for breast cancer. This finding is indeed supported by the expression pattern of the genes in our dataset. The LINC01234 gene shows a higher expression level in group 1 of breast cancer samples than in group 2 of breast cancer samples or normal samples. In contrast, the MET gene shows a lower expression level in group 1 of breast cancer samples than in group 2 of breast cancer samples or normal samples (Additional file 4). This results in a much larger *Δ*PCC(LINC01234, MET) for group 1 than for group 2 of breast cancer samples. The results of the analysis also agree with the reports by previous studies. LINC01234 is negatively related to miR-190b, and miR-190b is down-regulated in breast cancer [19], thus the expression level of LINC01234 can be higher in breast cancer. On the other hand, deregulation of MET is frequently observed in many types of cancer, including breast cancer [20].

### Possible effect of age and gender on survival and comparison of PCC and SCC

For each prognostic gene pair, we investigated possible effect of age and gender of cancer patients on their survival times using the Cox proportional hazards model. Unlike the hazard ratios (HR) associated with prognostic gene pairs, the hazard ratios associated with age were close to 1 for all gene pairs in all three cancer types (breast cancer, colon cancer and lung cancer). Thus, age is not a confounding factor. The hazard ratios associated with gender were in a wider range but with *p*-values >0.05, thus gender cannot be considered as a confounding factor, either. Detailed results for all prognostic gene pairs are available in Additional file 3.

PCC is known to be useful for detecting linear association but sensitive to outliers. Spearman’s rank correlation coefficient (SCC) also measures linear association like PCC, but is more robust to outliers than PCC because SCC is based on ranks instead of the actual observed values. For comparative purposes, we examined the scatter plots of all gene pairs in groups 1 and 2 of cancer samples and in normal samples from GTEx. No strictly linear association was observed in the prognostic gene pairs in breast cancer samples, but different association patterns were observed in normal samples (Additional file 5).

**Fig. 6:**
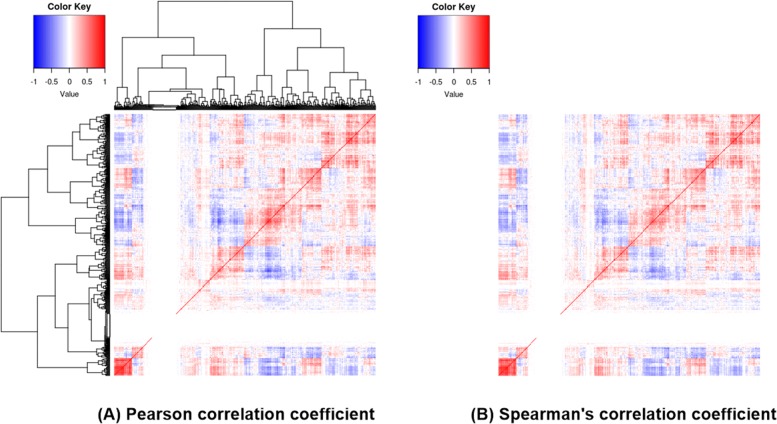
Heatmaps for correlation matrices in breast cancer. **a** Heatmap showing the Pearson correlation coefficient (PCC) between genes obtained by hierarchical clustering using the Euclidean distance measure and the ward.D2 linkage method. **b** Heatmap showing the Spearman correlation coefficient (SCC) between genes. The order of genes is the same for both heatmaps

For the direct comparison of PCC and SCC, we computed SCC between 516 genes in breast cancer and built a correlation matrix. Figure 6 shows two heatmaps built by average PCC and SCC in breast cancer samples. The order of genes is the same for both heatmaps. There is a difference in density between the two heatmaps but the distribution of density is quite similar to each other (enlarged heatmaps are available in Additional file 6). Furthermore, there is no significant difference in the top 10 prognostic gene pairs derived by PCC and SCC (Additional file 7).

### Comparison with other signatures and functional enrichment analysis in breast cancer

In a seminal paper, Venet et al. [21] showed that many random gene expression signatures are more predictive than known breast cancer signatures and that such random signatures are significantly associated with proliferation genes. More recently, Goh and Wong [22] also highlighted the problem of random signature superiority in breast cancer and other diseases. To address this issue, we compared our prognostic gene pairs with the meta-PCNA genes and noncancer signatures used in the study of Venet et al. [21]. The meta-PCNA genes are the genes that are most positively correlated with proliferation marker PCNA in normal tissues. The noncancer signatures are cancer irrelevant signatures such as those for predicting postprandial laughter on peripheral blood mononuclear cells, skin fibroblast localization, and social defeat in mice.

Eleven genes are shared by the 129 meta-PCNA genes and the 516 Malacards genes used in our study. Among the 147 prognostic gene pairs found in breast cancer, only a single gene pair IGKV1-5_MCM2 contains a meta-PCNA gene (MCM2) and no other prognostic gene pairs contain a meta-PCNA gene. Only one gene (TFAP2C) from noncancer signatures is included in our prognostic gene pair LINC01234_TFAP2C, and the remaining 146 prognostic gene pairs contain no noncancer signatures. These results indicate that the prognostic gene pairs found by our method in breast cancer are not associated with proliferation genes and that the prognostic gene pairs are not associated with noncancer signatures, either.

We also carried out functional enrichment analysis of the genes involved in the prognostic gene pairs for breast cancer using DAVID (https://david.ncifcrf.gov). From the analysis, we found four clusters with high enrichment scores (Table 4). For comparison, we performed the log-rank test with the 404 genes in the noncancer signatures, and derived 31 significant gene pairs (Additional file 8), which correspond to those with the top 25% *Δ*PCC. In functional enrichment analysis of the 44 genes included in the 31 gene pairs from noncancer signatures, no meaningful clusters were derived. Detailed results of the functional enrichment analysis are available in Additional file 9.

**Table 4:** Functional enrichment analysis of the genes in the prognostic gene pairs for breast cancer

		#genes	*p*-value	FDR
Annotation cluster 1	Enrichment score: 13.55			
KEGG_PATHWAY	hsa05200:Pathways in cancer	36	1.64E-22	1.98E-19
KEGG_PATHWAY	hsa05215:Prostate cancer	15	1.40E-12	1.70E-09
KEGG_PATHWAY	hsa04151:PI3K-Akt signaling pathway	19	9.74E-08	1.18E-04
Annotation cluster 2	Enrichment score: 7.93			
KEGG_PATHWAY	hsa04510:Focal adhesion	18	2.31E-10	2.80E-07
KEGG_PATHWAY	hsa05205:Proteoglycans in cancer	16	1.13E-08	1.37E-05
KEGG_PATHWAY	hsa04015:Rap1 signaling pathway	16	2.20E-08	2.66E-05
KEGG_PATHWAY	hsa05100:Bacterial invasion of epithelial cells	10	3.23E-07	3.91E-04
Annotation cluster 3	Enrichment score: 6.62			
UP_KEYWORDS	Growth factor	11	7.37E-09	9.42E-06
GOTERM_MF_DIRECT	GO:0008083 growth factor activity	12	2.54E-08	3.52E-05
GOTERM_BP_DIRECT	GO:0050679 positive regulation of epithelial cell proliferation	6	7.40E-05	1.25E-01
Annotation cluster 4	Enrichment score: 5.44			
GOTERM_MF_DIRECT	GO:0044212 transcription regulatory region DNA binding	13	4.59E-08	6.36E-05
GOTERM_BP_DIRECT	GO:0045893 positive regulation of transcription, DNA-templated	17	9.99E-07	1.69E-03
GOTERM_BP_DIRECT	GO:0045892 negative regulation of transcription, DNA-templated	12	1.03E-03	1.73E-00

### Prognostic gene pairs in colon cancer and lung cancer

**Fig. 7:**
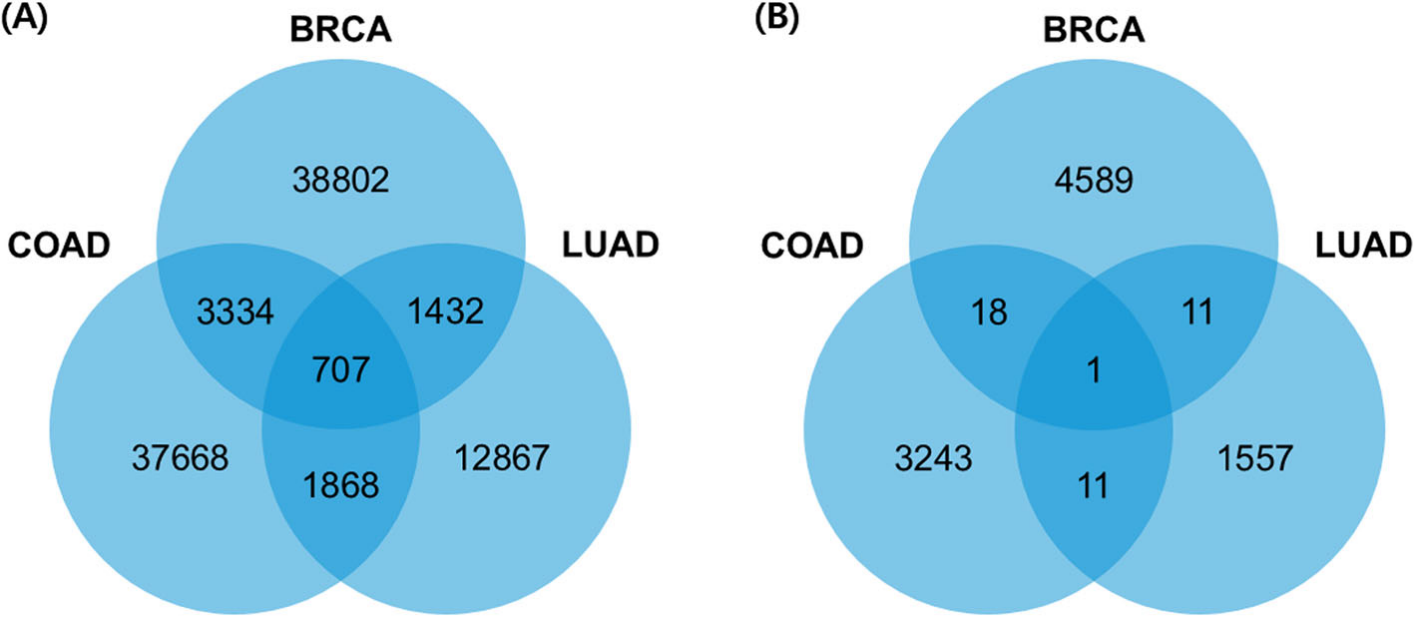
The number of prognostic gene pairs shared by three cancer types. **a** The number of prognostic gene pairs without any constraints. **b** The number of prognostic gene pairs with a *p*-value of the log-rank test <0.05. The single gene pair shared by the three cancer types is CCNB1_TERT

From the log-rank test with 43,577 gene pairs in colon cancer and 16,874 gene pairs in lung cancer, we found 38 and 12 prognostic gene pairs for colon cancer and lung cancer, respectively. We examined whether prognostic gene pairs are shared by the three cancer types (breast cancer, colon cancer and lung cancer) (Fig. 7). The Venn diagram in Figure 7a compares all gene pairs without any constraints, and the Venn diagram in Figure 7b compares the number of gene pairs with a *p*-value of the log-rank test <0.05. Among the significant prognostic gene pairs, the gene pair CCNB1_TERT is the only prognostic gene pair shared by the three types of cancer.

CCNB1 has been known to be associated with breast cancer [23], but so far there is little or no direct relation known between CCNB1 and TERT. Unlike most normal cells where there is lack of telomerase activity, upregulation of TERT transcriptional activity is detected in 80-90% of tumor cells [24–26]. TERT is known to repress the cell cycle regulator p21 in cancer [24], and p21 itself in tern inhibits several p53-dependent genes (CCNB1 is one of the p53-dependent genes) [23]. Thus, when the p21 level is decreased by TERT in cancer cells, the expression level of CCNB1 can be increased because CCNB1 is no longer inhibited by p21.

To investigate whether such relation exists in the dataset used in our study, we re-examined the expression levels of the genes and their *Δ*PCCs in three types of cancer. Both CCNB1 and TERT consistently showed higher expression levels in cancer tissues of three types than in normal tissues (Additional file 10). This result supports the known fact that TERT expression is increased in tumor cells and that CCNB1 expression is also increased because its repressor p21 is inhibited by TERT. In addition to this, group 1 of cancer tissues showed a higher *Δ*PCC than group 2 of cancer tissues. Note that in our survival analysis, group 1 is a group of cancer samples with a big change in PCC of a gene pair (i.e., top 25% *Δ*PCC) from the normal samples, and group 2 is a group of the remaining cancer samples. The Kaplan-Meier plot in Addition file 10 shows that group 1 has a lower survival rate than group 2 in both breast cancer and lung cancer. However, the opposite is observed in colon cancer. The reason for the lower survival rate of group 2 of colon cancer samples with respect to CCNB1_TERT can be explained by the expression pattern of TERT. As shown in Addition file 10, the expression pattern of TERT in colon cancer is very different from that in breast cancer and lung cancer. Unlike in breast cancer and lung cancer, TERT shows increased expression levels in group 2 of colon cancer samples, which is associated with the lower survival rate of group 2 of colon cancer samples. Relevant data are given in Additional file 10.

The survival rates of two groups of patients with respect to the prognostic gene pairs for colon cancer and lung cancer are shown in Fig. 3b and Fig. 3c, respectively. All prognostic gene pairs found for colon cancer and lung cancer are available in Additional file 3.

**Fig. 8:**
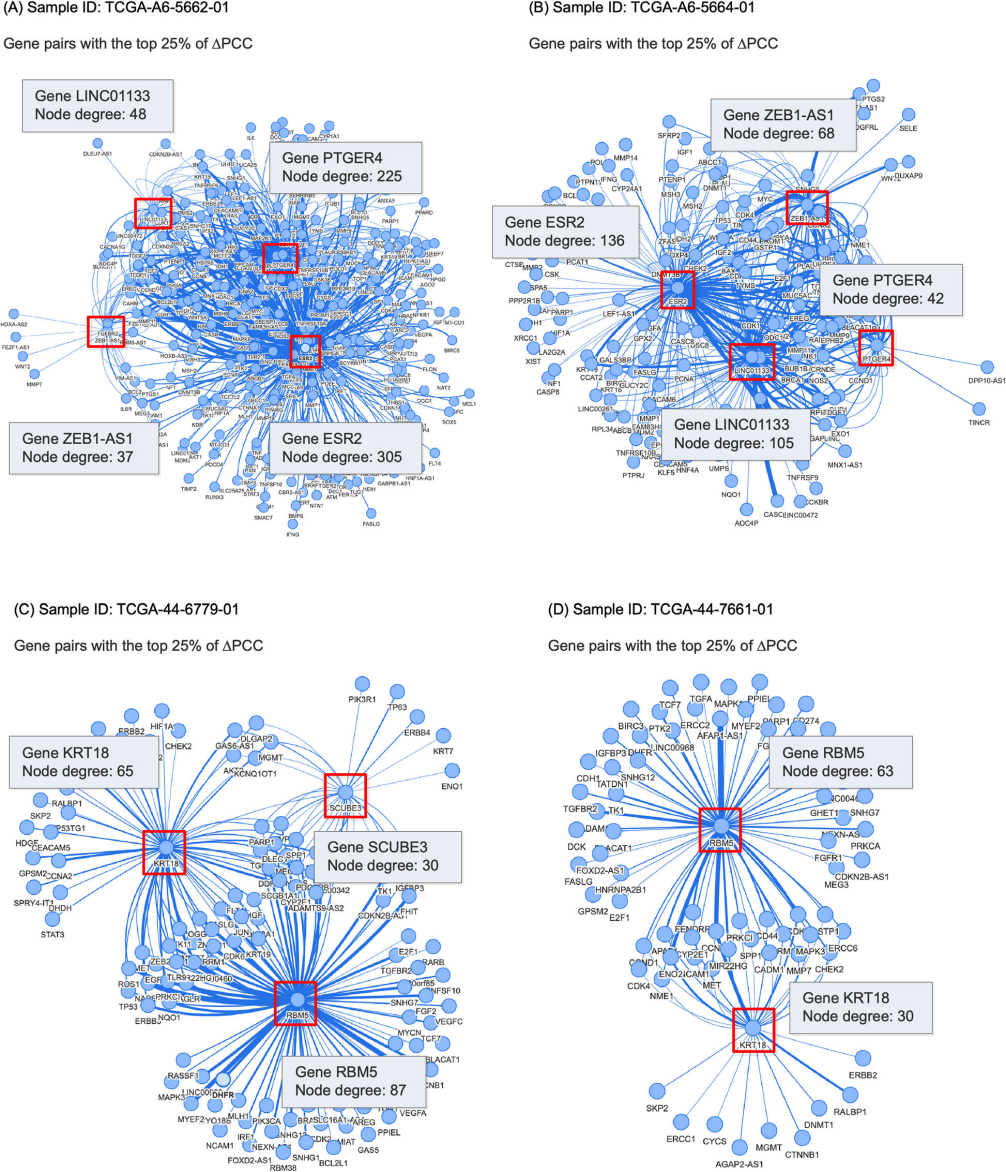
Example of colon and lung cancer patient-specific networks. Samples **a** and **b** (ID: TCGA-A6-5662-01 and TCGA-A6-5664-01) are colon cancer samples and samples **c** and **d** (ID: TCGA-44-6779-01 and TCGA-44-7661-01) are lung cancer samples. Samples **a** and **c** belong to group 1 (gene pairs with the highest 25% of *Δ*PCC) in the log-rank test and samples **b** and **d** belong to group 2. The networks are available at http://bclab.inha.ac.kr/pancancer

Figure 8 shows examples of patient-specific networks for the two types of cancer. In Figure 8, samples A and B are colon cancer samples and samples C and D are lung cancer samples. Samples A and C belong to group 1 in the survival analysis (i.e., they show a big change in PCC of prognostic gene pairs from normal samples) and samples B and D belong to group 2. Two prognostic gene pairs for colon cancer, LINC01133_PTGER4 and ESR2_ZEB1-AS1, are included in the network for colon cancer sample A but are missing in the network for another colon cancer sample B. Likewise, two prognostic gene pairs for lung cancer, KRT18_SCUBE3 and KRT18_RBM5, are included in the network for lung cancer sample C but are missing in the network for another lung cancer sample D.

A similar expression pattern was observed in the prognostic gene pair LINC01133_ PTGER4 for colon cancer. As shown Additional file 4, the average expression level of LINC01133 of group 1 of colon cancer samples is higher than that of group 2 of colon cancer samples or normal samples. In contrast, the average expression level of PTGER4 of group 1 of colon cancer samples is lower than that of group 2 of colon cancer samples or normal samples. Therefore, group 1 of colon samples shows a larger *Δ*PCC(LINC01133, PTGER4) than group 2 of colon samples.

Both LINC01133 and PTGER4 genes are related with p21, which is a well-known tumor-suppressor gene. PTGER4 (EP4) induces expression of p21, whereas LINC01133 is negatively correlated with p21 [27, 28]. Thus, the low expression of PTGER4 in group 1 and the high expression of LINC01133 are associated with the decreased expression of p21 in group 1, which in turn is associated with the lower survival time of group 1 than group 2.

We also investigated the genes in the prognostic gene pairs KRT18_SCUBE3 and KRT18_RBM5 for lung cancer. KRT18 shows a higher expression level in group 1 of lung cancer samples than in group 2 of lung cancer samples and in normal samples. In contrast, both SCUBE3 and RBM5 genes show a lower expression level in group 1 of lung cancer samples than in group 2 of lung cancer samples and in normal samples. Thus, both gene pairs KRT18_SCUBE3 and KRT18_RBM5 have negative correlations. Furthermore, the gene pairs have a larger *Δ*PCC in group 1 than in group 2 of lung cancer samples. These results are consistent with the previous report that high expression levels of KRT18 are correlated with unfavorable survival of lung cancer patients [29].

The results indicate that prognosis of individual cancer patients is associated with the existence of prognostic gene pairs in the patient-specific network and the size of the subnetwork of prognostic gene pairs in the patient-specific network.

## Conclusion

In this paper, we proposed a new method for constructing patient-specific gene networks and for finding prognostic gene pairs for three types of cancer, breast invasive carcinoma, colon adenocarcinoma and lung adenocarcinoma. The key difference of our method from previous ones is that (1) it is intended for finding prognostic gene pairs rather than prognostic genes and (2) gene pairs are more reliable prognostic signatures than individual genes because prognostic gene pairs can be found even in gene expression profiles where no significant prognostic genes exist. For breast invasive carcinoma, colon adenocarcinoma, and lung adenocarcinoma, we found a total of 147, 38, and 12 potential prognostic gene pairs, respectively.

The prognostic gene pairs found in our study show no association with age or gender of cancer patients. They are not correlated with proliferation genes, which are known to confound the predictive power of random signatures. Evaluation of our method with extensive data sets of three cancer types showed that our approach is general and that gene pairs can serve as more reliable prognostic signatures for cancer than individual genes. We also found that prognosis of individual cancer patients is associated with the existence of prognostic gene pairs in the patient-specific network and the size of the patient-specific network.

Although preliminary, our approach will be useful for finding gene pairs to predict survival time of patients and to tailor treatments to individual characteristics. The program for dynamically constructing patient-specific gene networks and for finding prognostic gene pairs is available at http://bclab.inha.ac.kr/pancancer.

## Supplementary information

**Additional file 1** A total of 516 genes for BRCA, 466 genes for COAD, and 410 genes for LUAD are listed in **BRCA.csv**, **COAD.csv**, and **LUAD.csv**, respectively.

**Additional file 2** Clustering of cancer samples with respect to gene pairs in the log-rank test.

**Additional file 3** Results of the log-rank test and the Cox proportional hazards model.

**Additional file 4** Expression levels of top prognostic gene pairs in three cancer types.

**Additional file 5** Scatter plots of the expression levels (on log2 scale) of genes in the potential prognostic gene pairs of breast cancer. Genes that were not expressed are excluded in the scatter plots.

**Additional file 6** Heatmaps for correlation matrices by Pearson correlation coefficient (PCC) and Spearman’s correlation coefficient (SCC) in three cancer types.

**Additional file 7** Top 10 prognostic gene pairs derived by PCC and SCC in three cancer types.

**Additional file 8** Significant gene pairs derived from noncancer signatures used in the study of venet et al. [21].

**Additional file 9** The results of functional enrichment analysis of the genes involved in the 147 prognostic gene pairs for BRCA.

**Additional file 10** Additional analysis of the prognostic gene pair CCNB1_TERT, the only prognostic gene pair common to BRCA, COAD and LUAD

## Data Availability

Additional files are available at http://bclab.inha.ac.kr/pancancer.

## Abbreviations

BRCA: Breast invasive carcinoma
COAD: Colon adenocarcinoma
FDR: False discovery rate
GTEx: Genotype-tissue expression
HR: Hazard ratio
lncRNA: Long non-coding RNA
LUAD: Lung adenocarcinoma
PCC: Pearson correlation coefficient
SCC: Spearman’s rank correlation coefficient
TCGA: The cancer genome atlas
