# Supplementary material for: Finding prognostic gene pairs for cancer from patient-specific gene networks
Source: BMC Med Genomics. 2019 Dec 20;12(Suppl 8):179. doi: 10.1186/s12920-019-0634-0 (PMC6923916; doi:10.1186/s12920-019-0634-0)
Supplement: Supplementary file 2 — Additional file 2 Clustering of cancer samples with respect to gene pairs in the log-rank test. [file 12920_2019_634_MOESM2_ESM.pdf]

Due to file size limit, additional file 2 is available at http://bclab.inha.ac.kr/pancancer.
