# Supplementary figures and images for: Finding prognostic gene pairs for cancer from patient-specific gene networks
Source: BMC Med Genomics. 2019 Dec 20;12(Suppl 8):179. doi: 10.1186/s12920-019-0634-0 (PMC6923916; doi:10.1186/s12920-019-0634-0)

Additional file 4

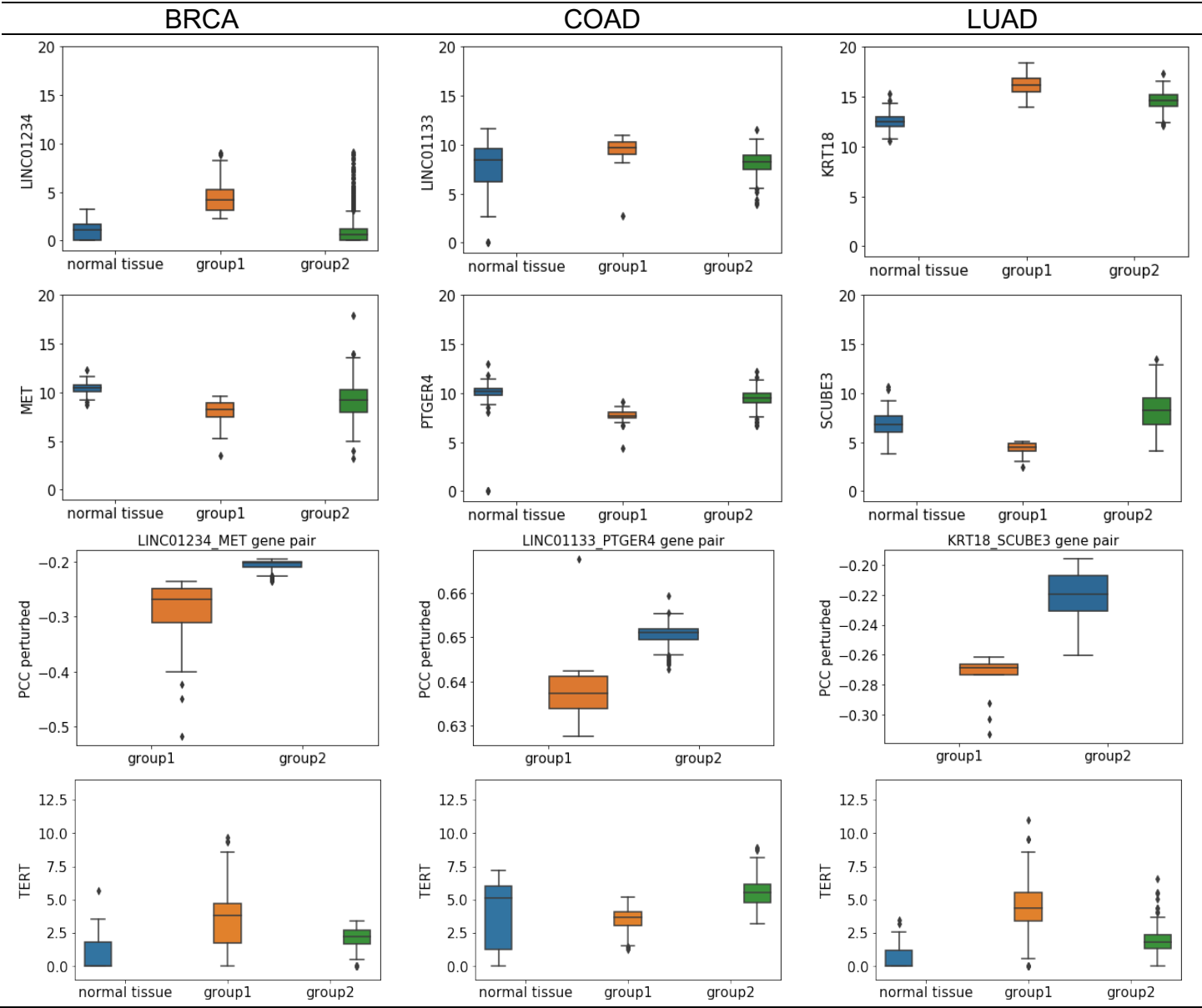

Supplement: Supplementary file 4 — Additional file 4 Expression levels of top prognostic gene pairs in three cancer types. [file 12920_2019_634_MOESM4_ESM.pdf]

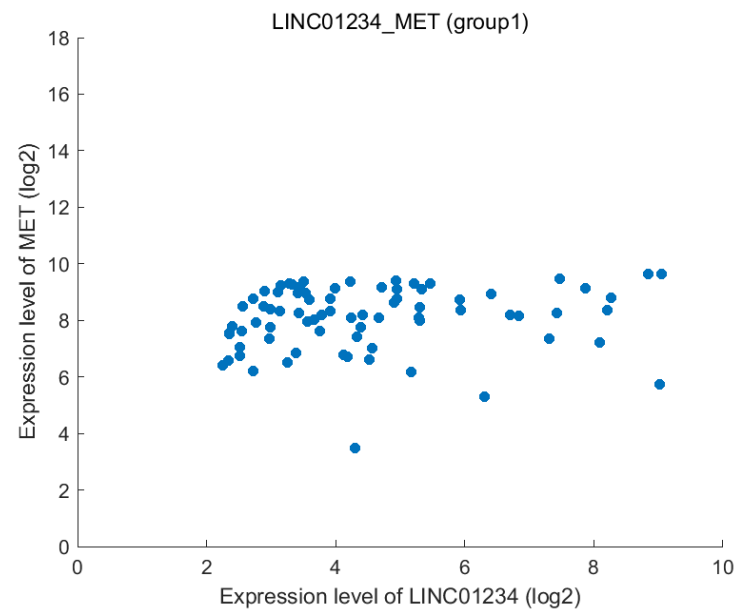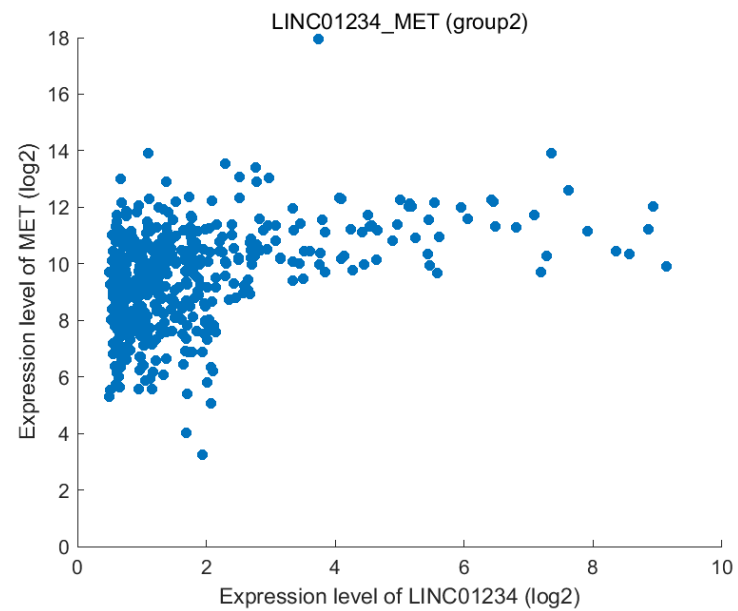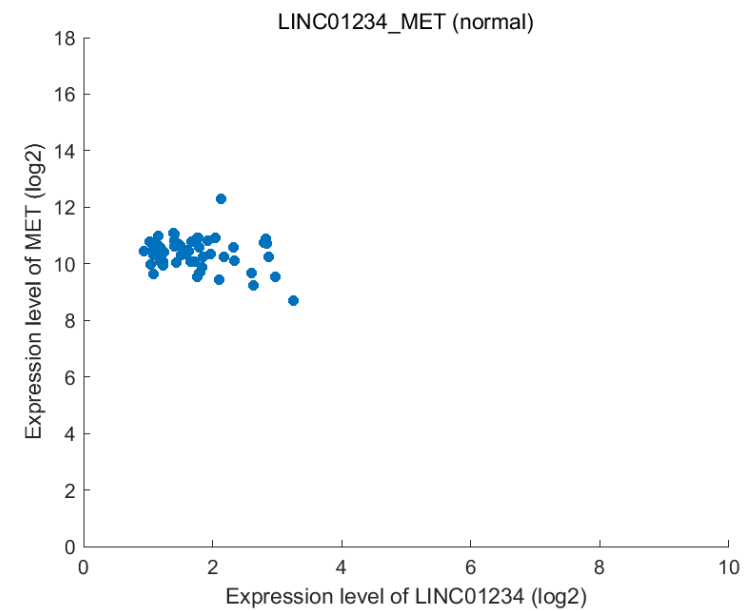

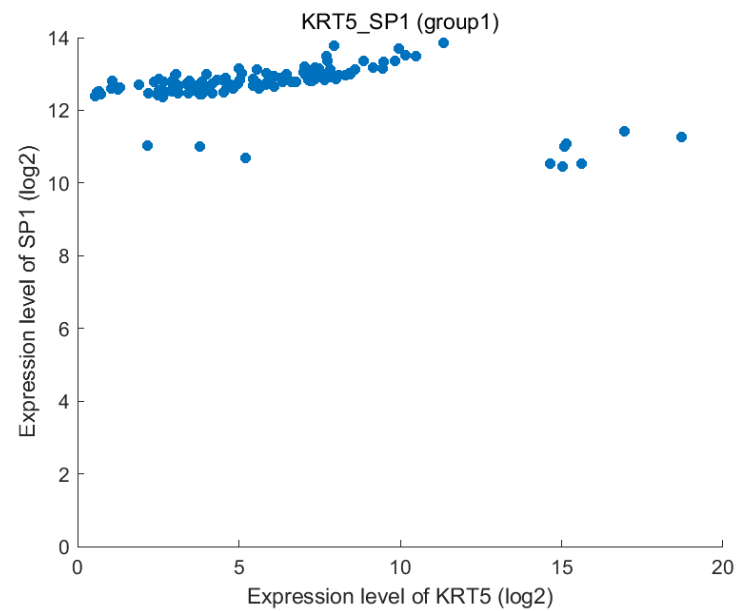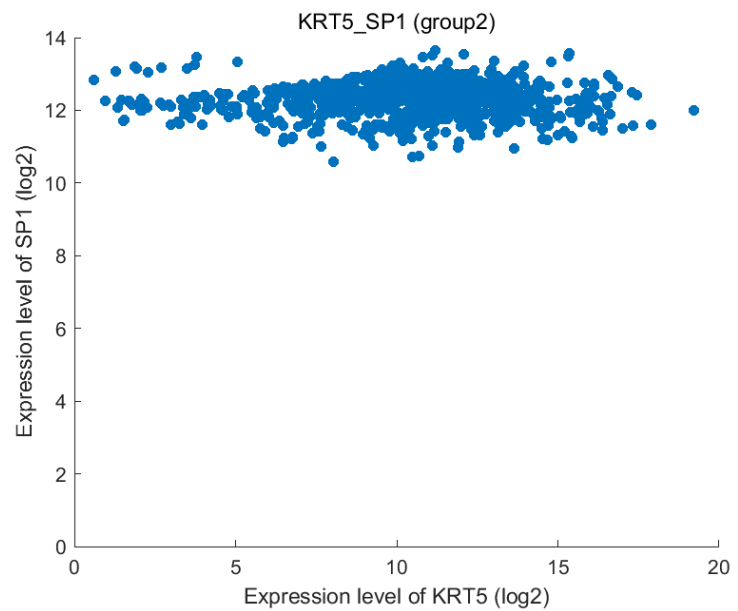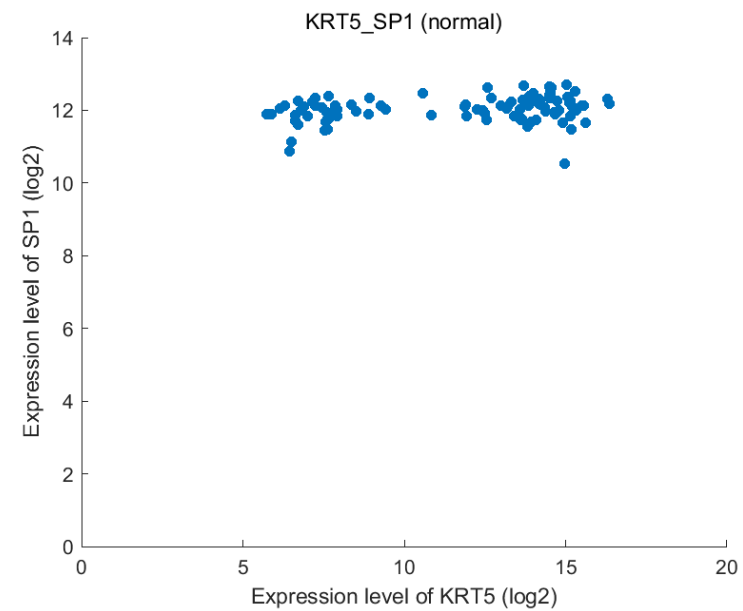

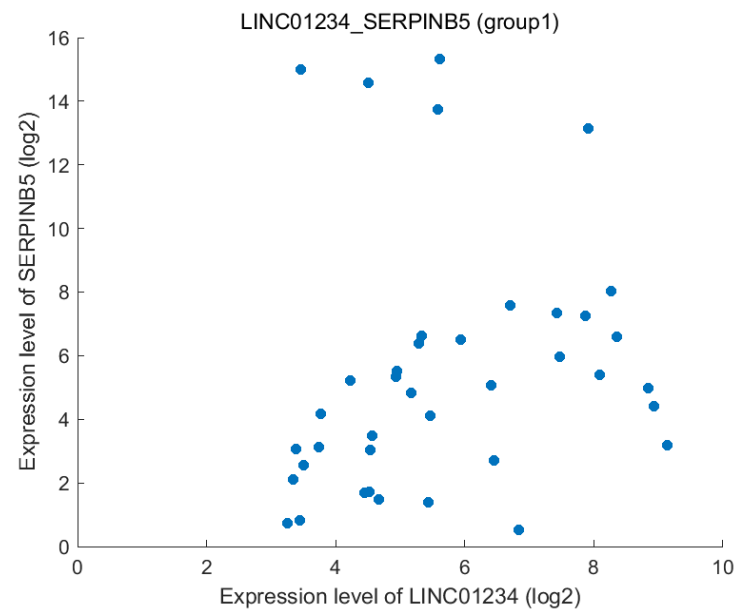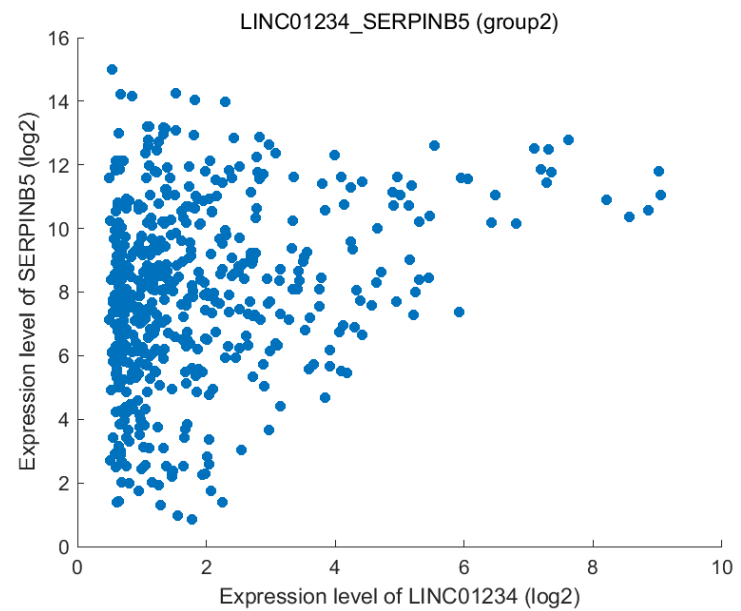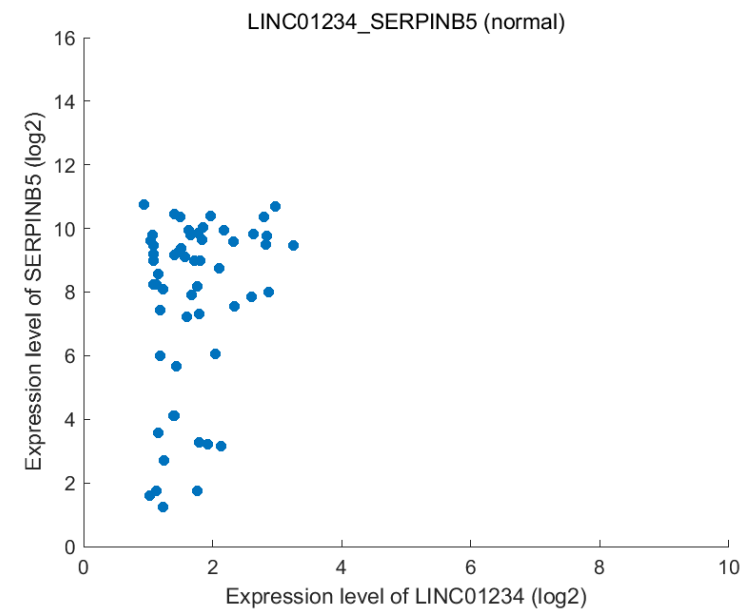

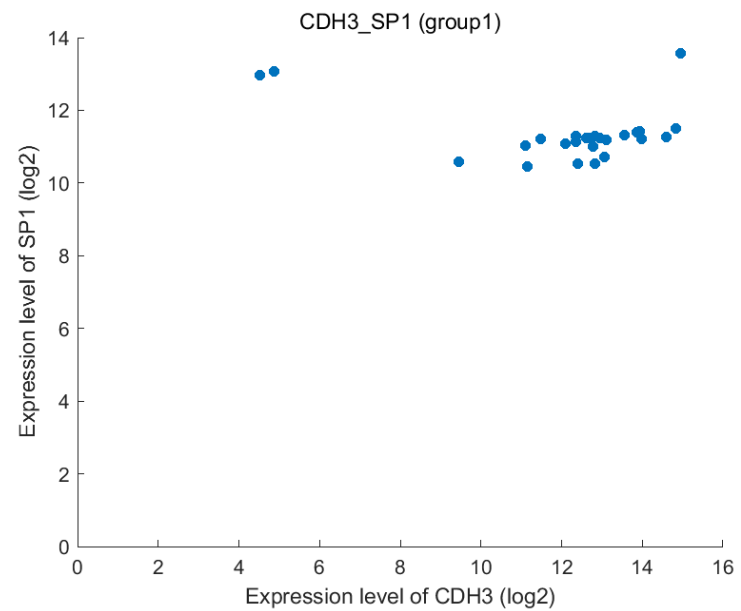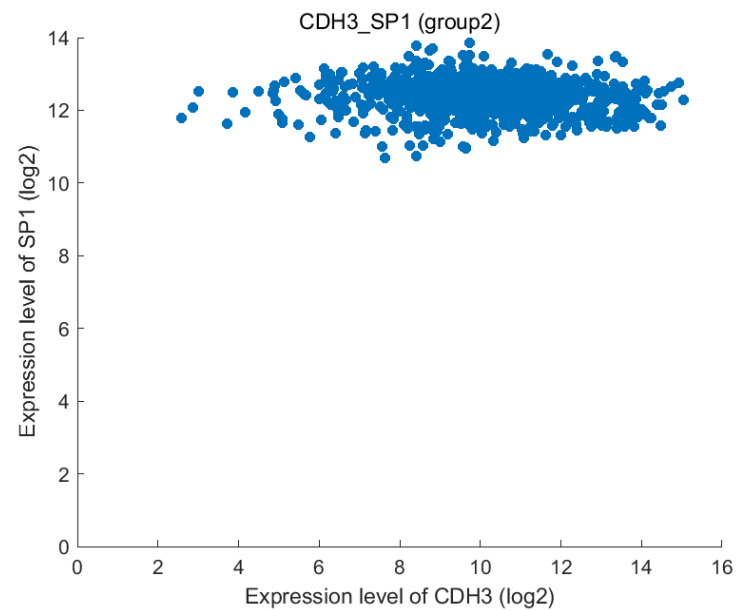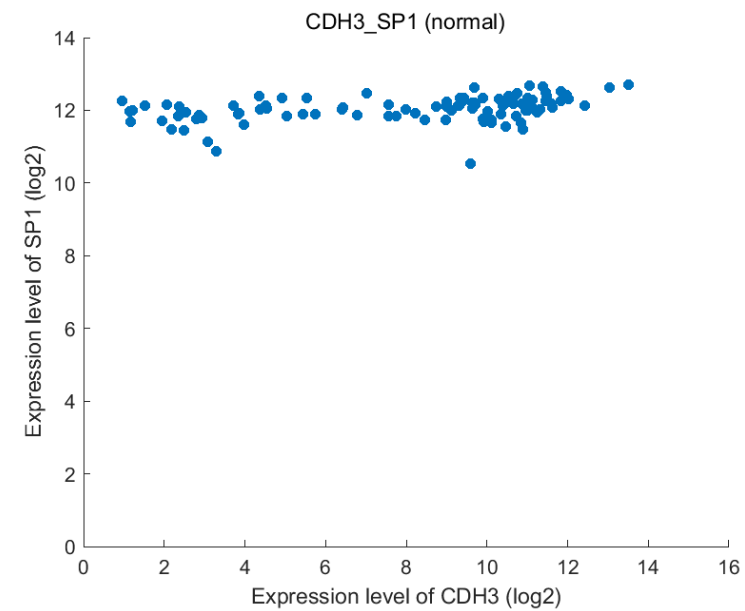

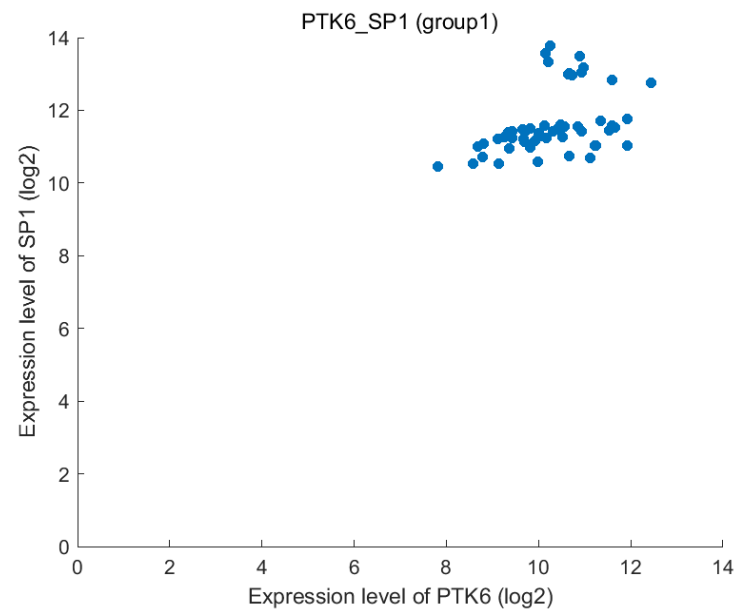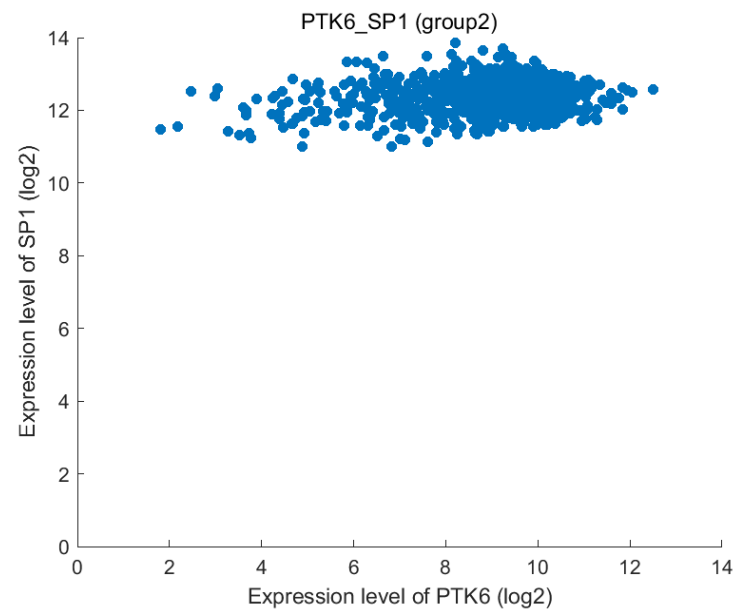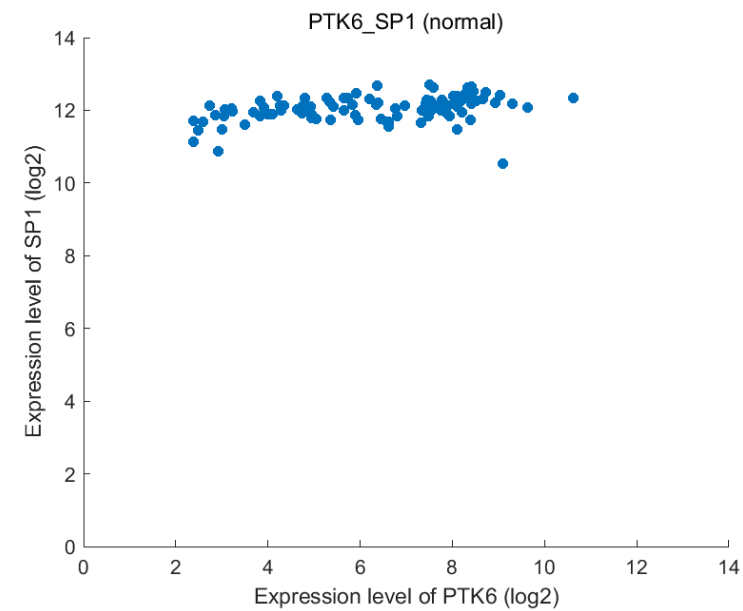

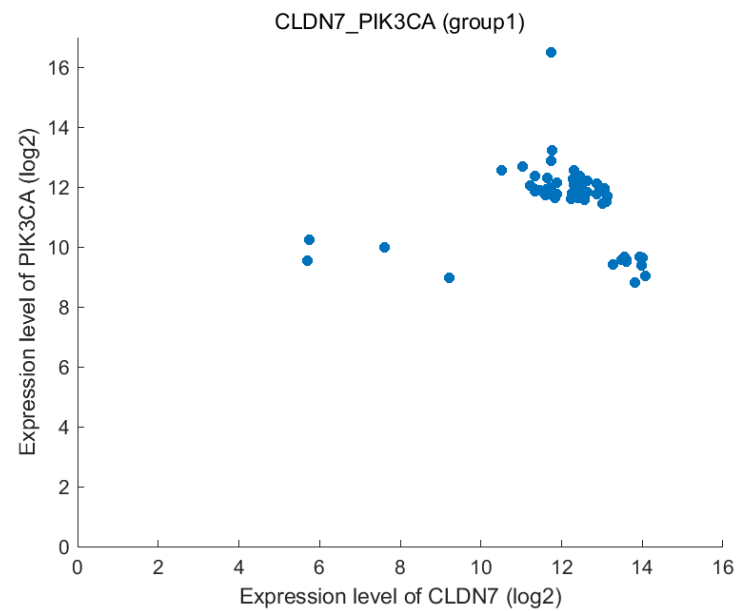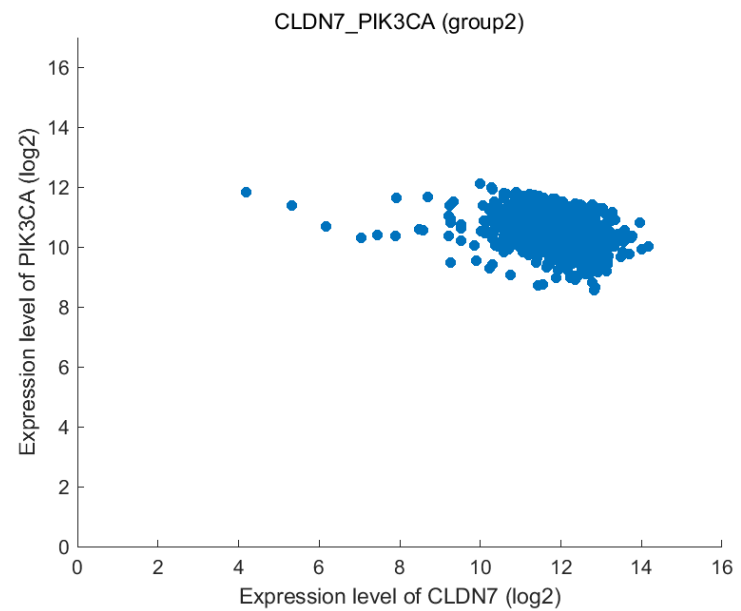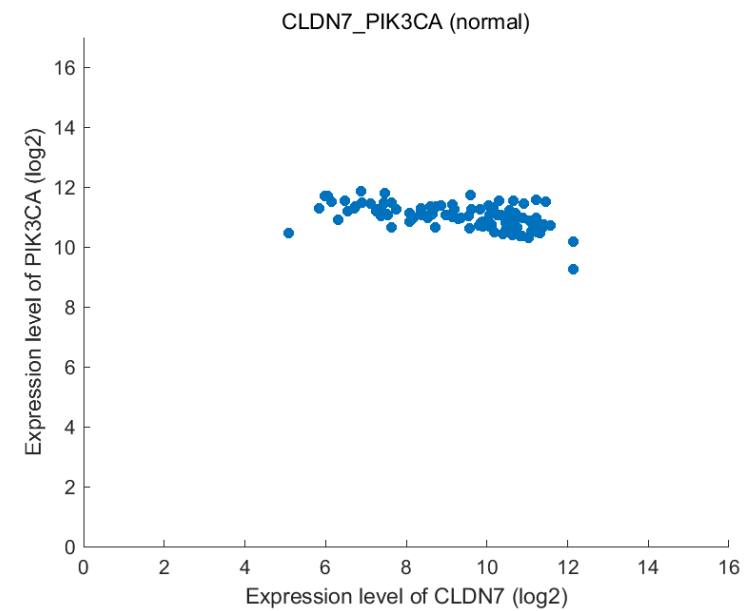

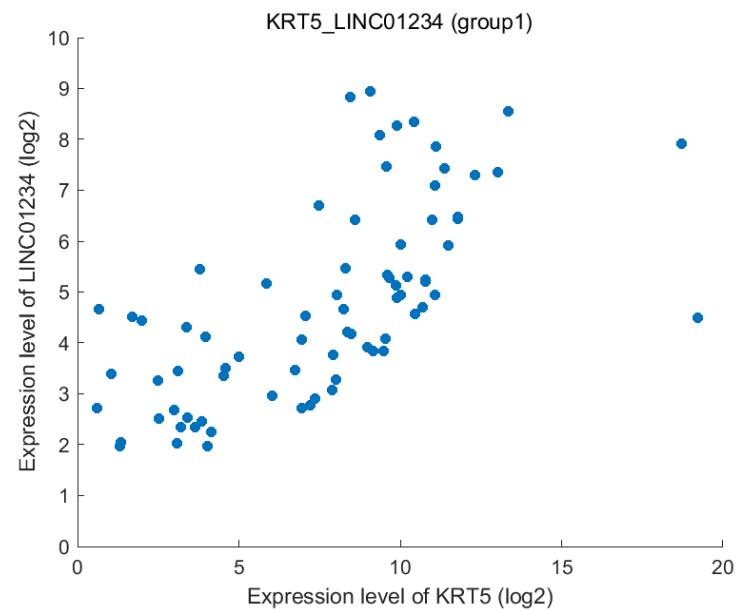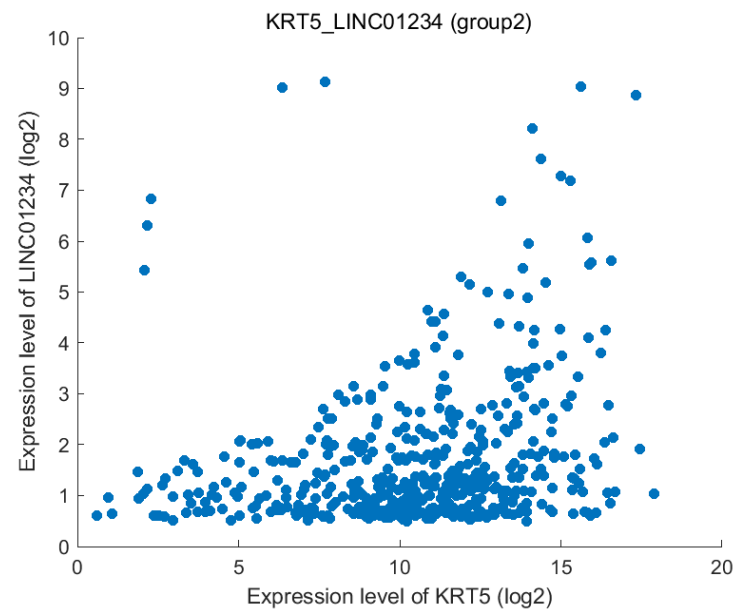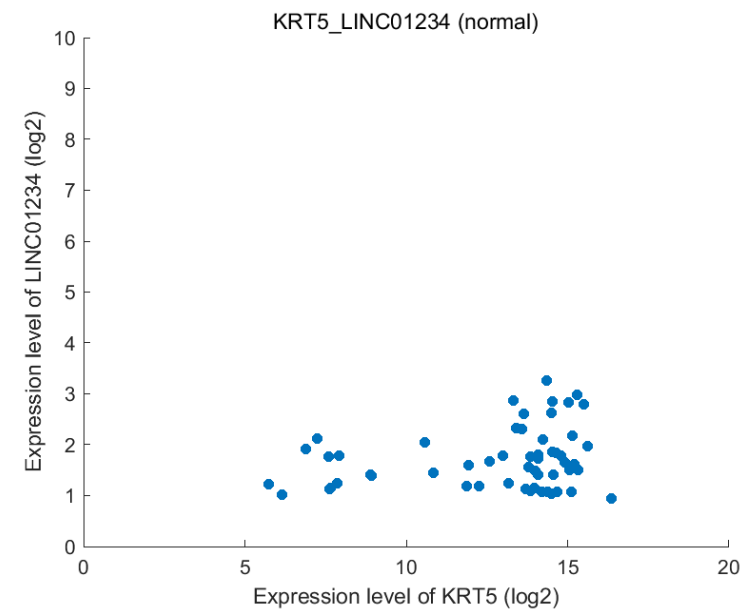

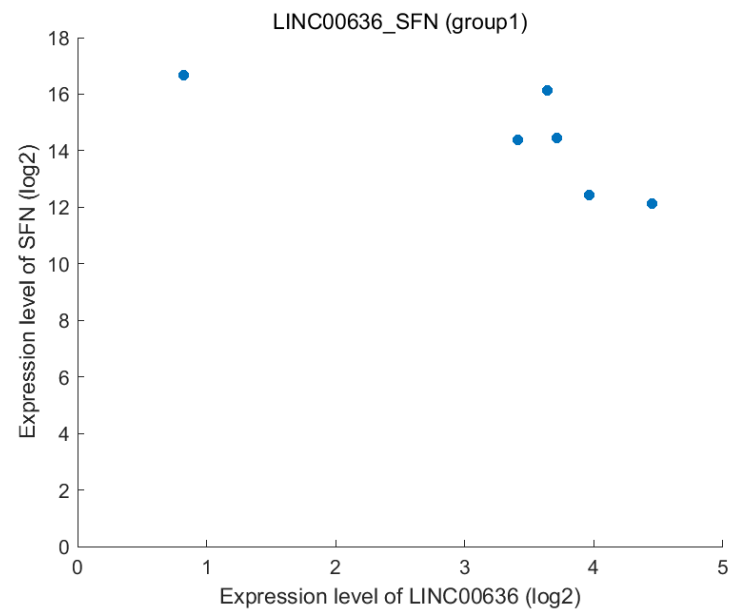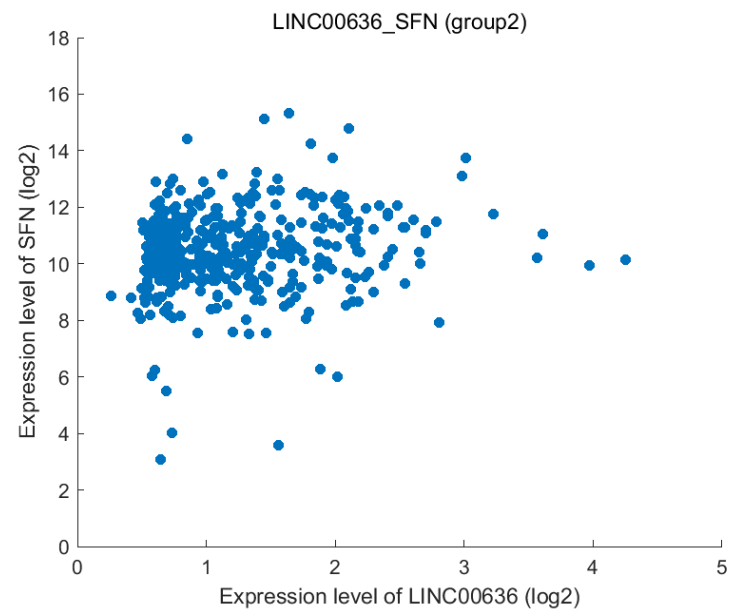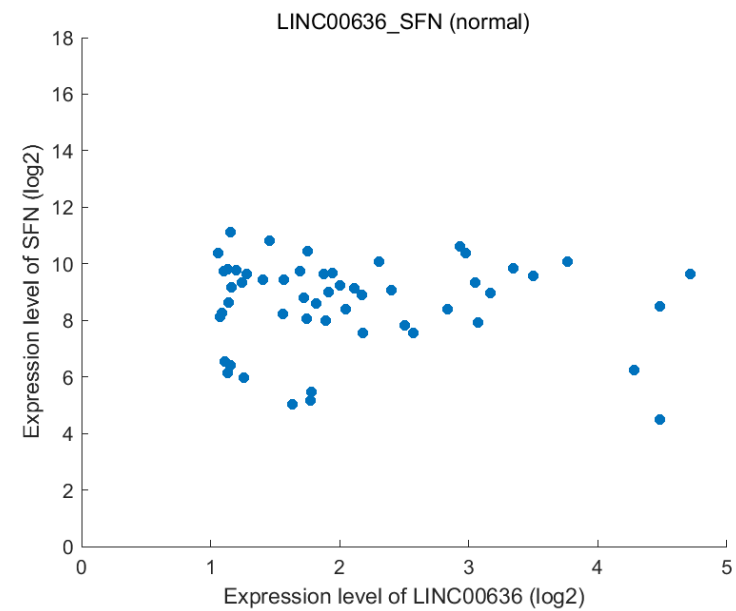

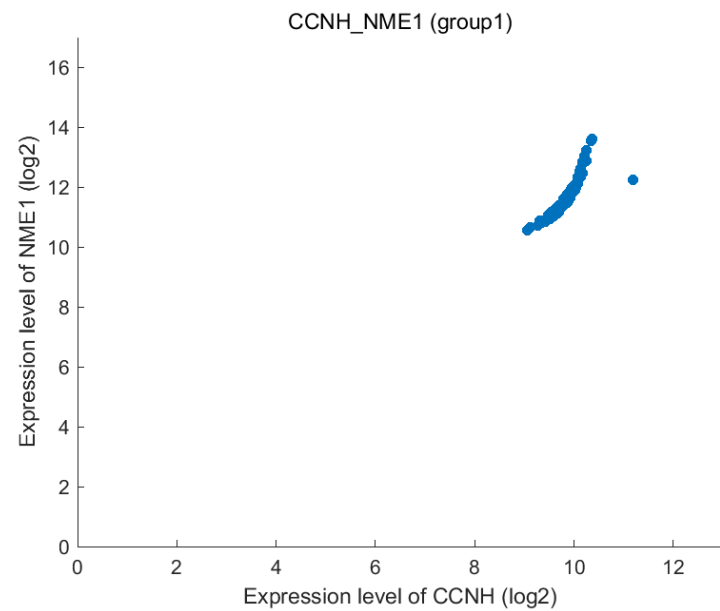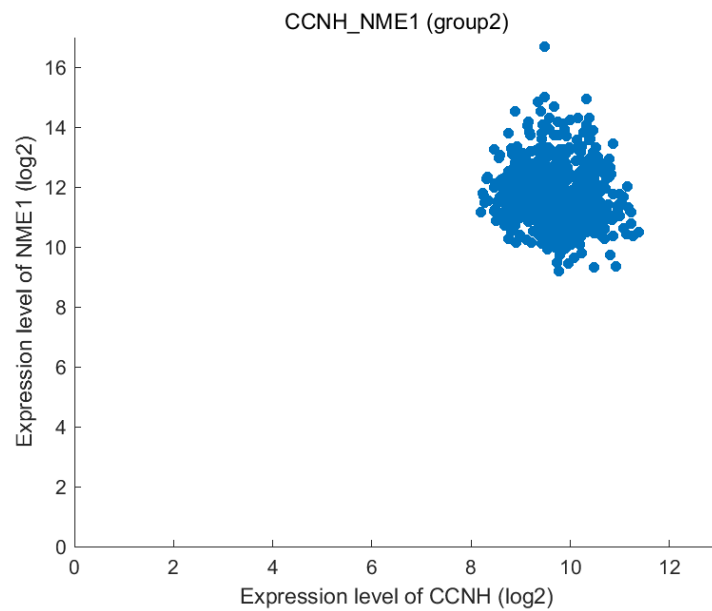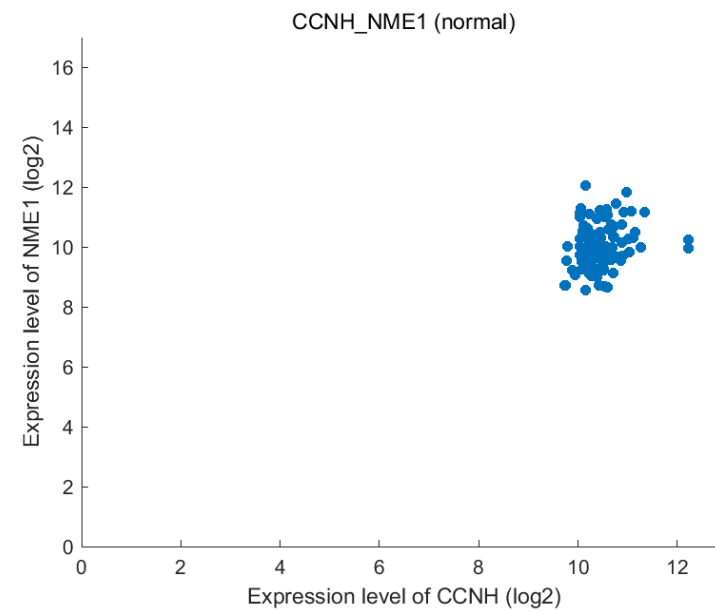

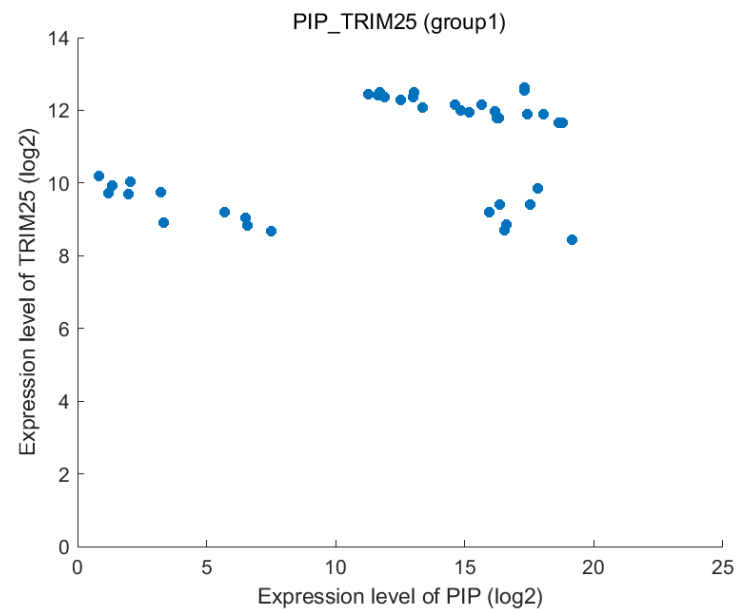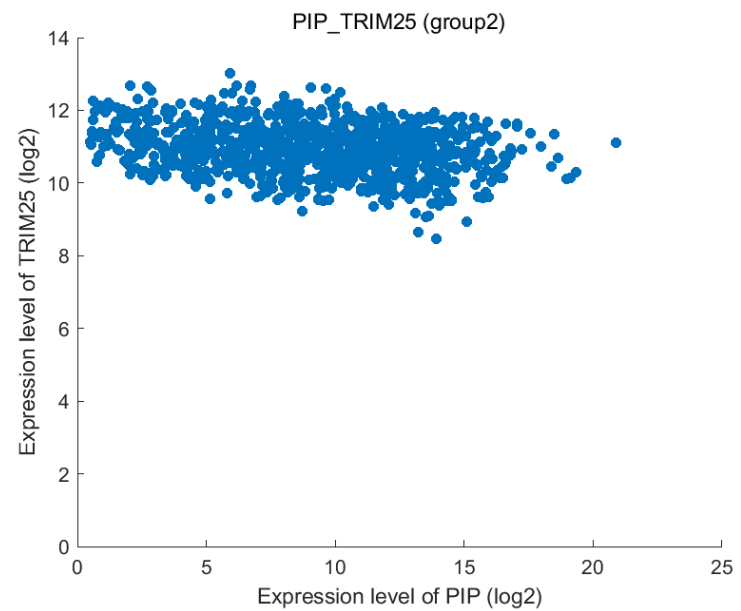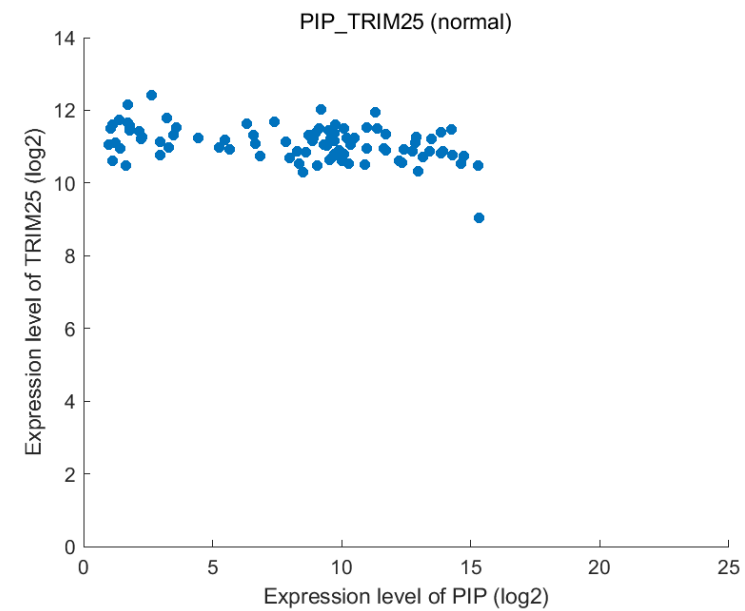

Supplement: Supplementary file 5 — Additional file 5 Scatter plots of the expression levels (on log2 scale) of genes in the potential prognostic gene pairs of breast cancer. Genes that were not expressed are excluded in the scatter plots. [file 12920_2019_634_MOESM5_ESM.pdf]

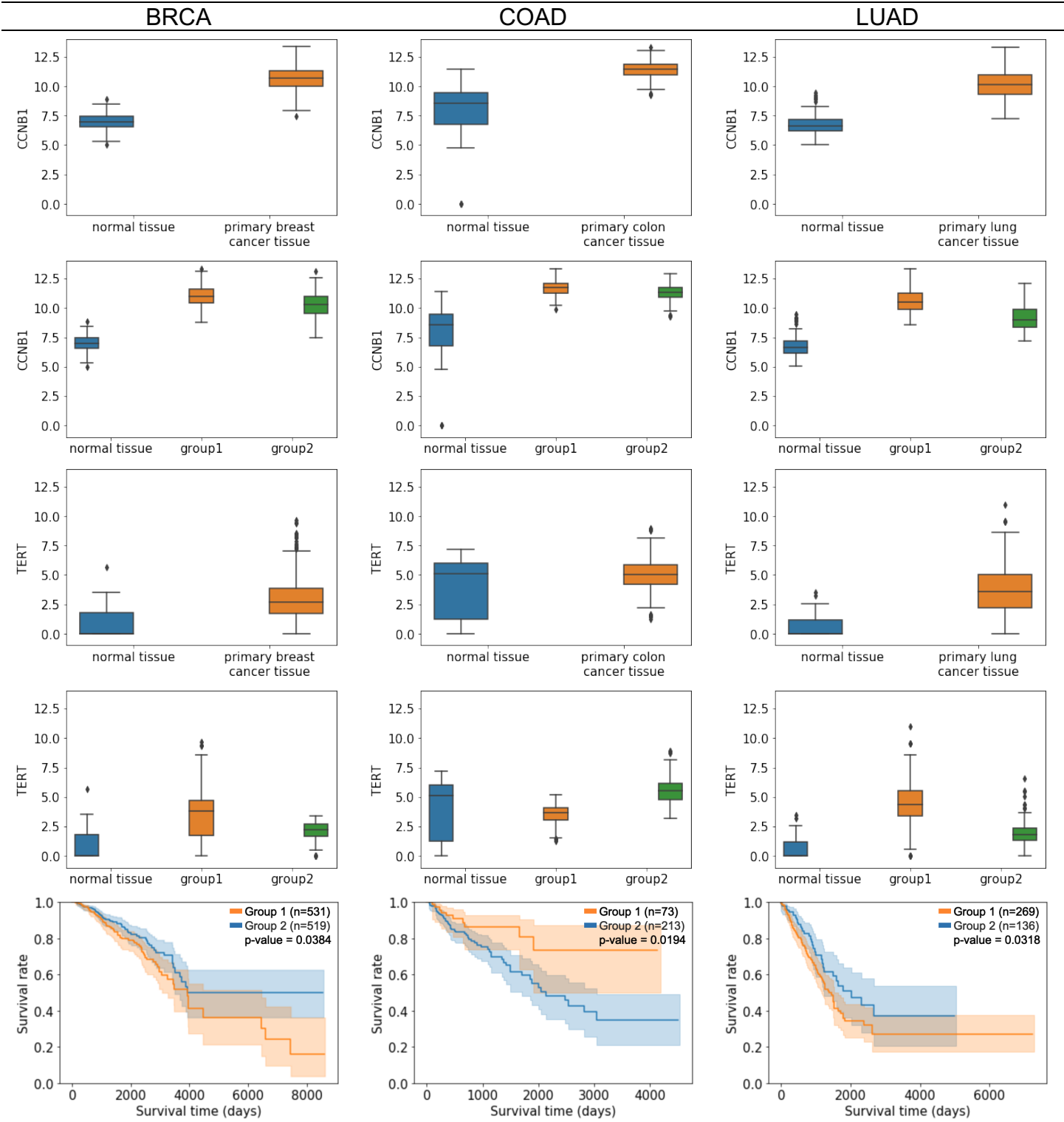

Supplement: Supplementary file 10 — Additional file 10 Additional analysis of the prognostic gene pair CCNB1_TERT, the only prognostic gene pair common to BRCA, COAD and LUAD [file 12920_2019_634_MOESM10_ESM.pdf]
