# Supplementary material for: Finding prognostic gene pairs for cancer from patient-specific gene networks
Source: BMC Med Genomics. 2019 Dec 20;12(Suppl 8):179. doi: 10.1186/s12920-019-0634-0 (PMC6923916; doi:10.1186/s12920-019-0634-0)

BRCA

(A) Pearson correlation coefficient

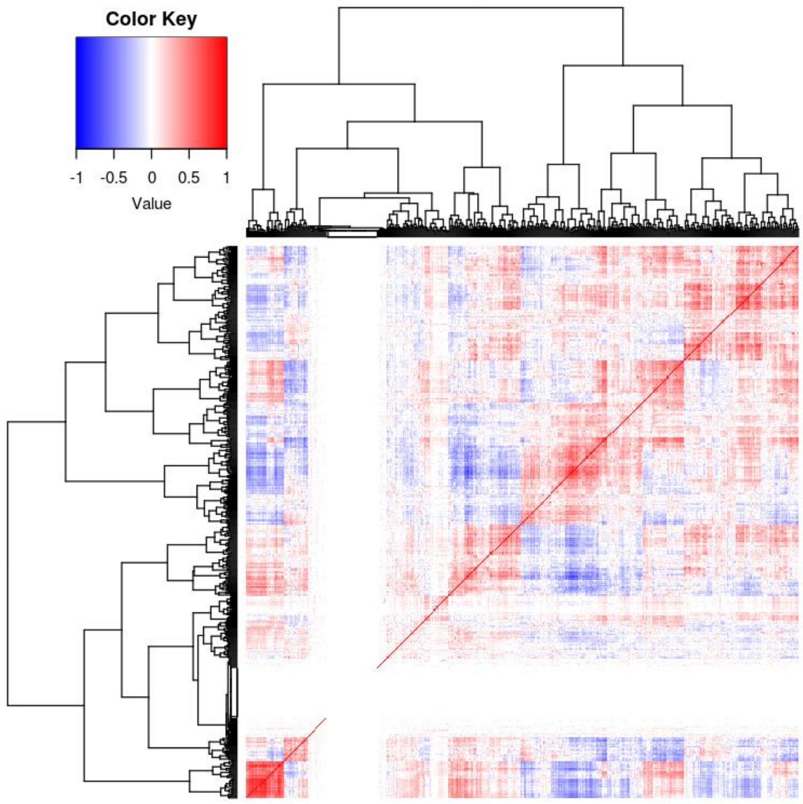

(B) Spearman's correlation coefficient

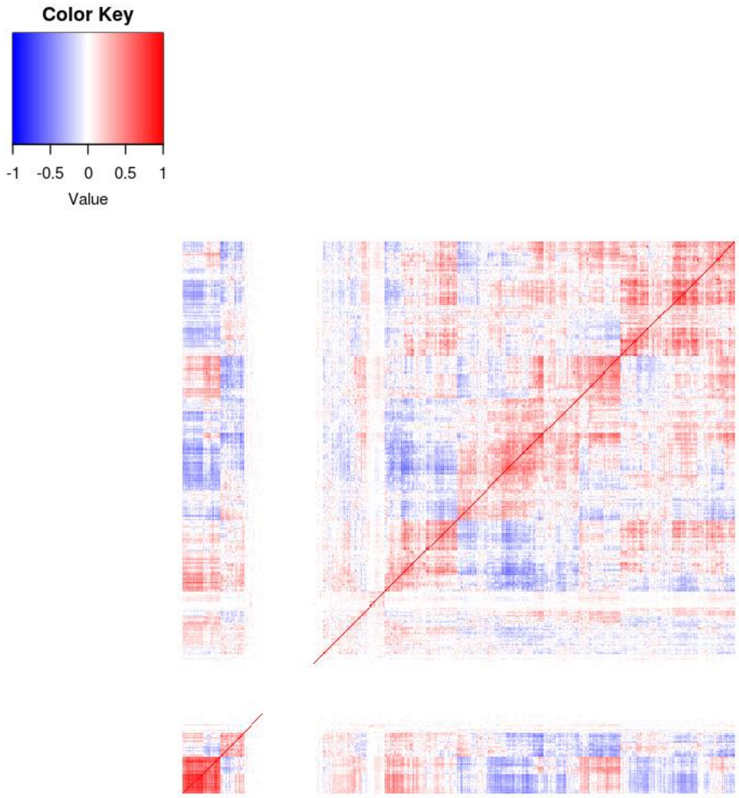

COAD

(A) Pearson correlation coefficient

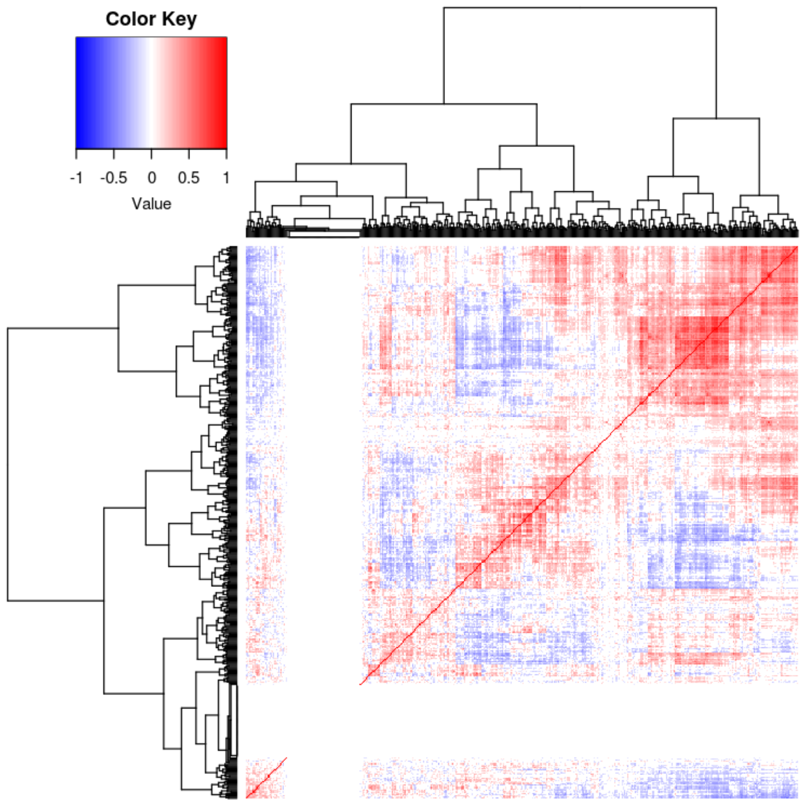

(B) Spearman's correlation coefficient

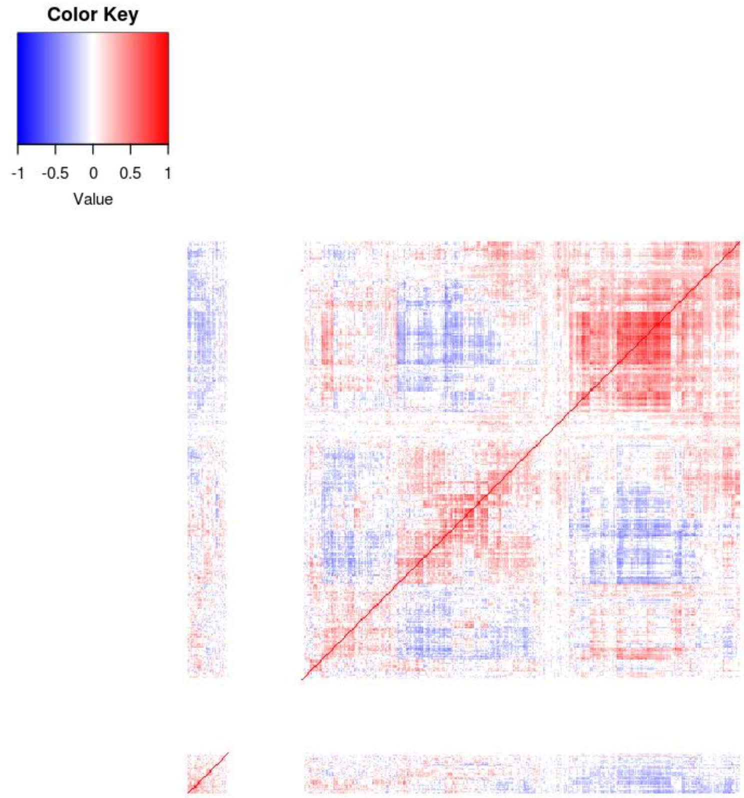

LUAD

(A) Pearson correlation coefficient

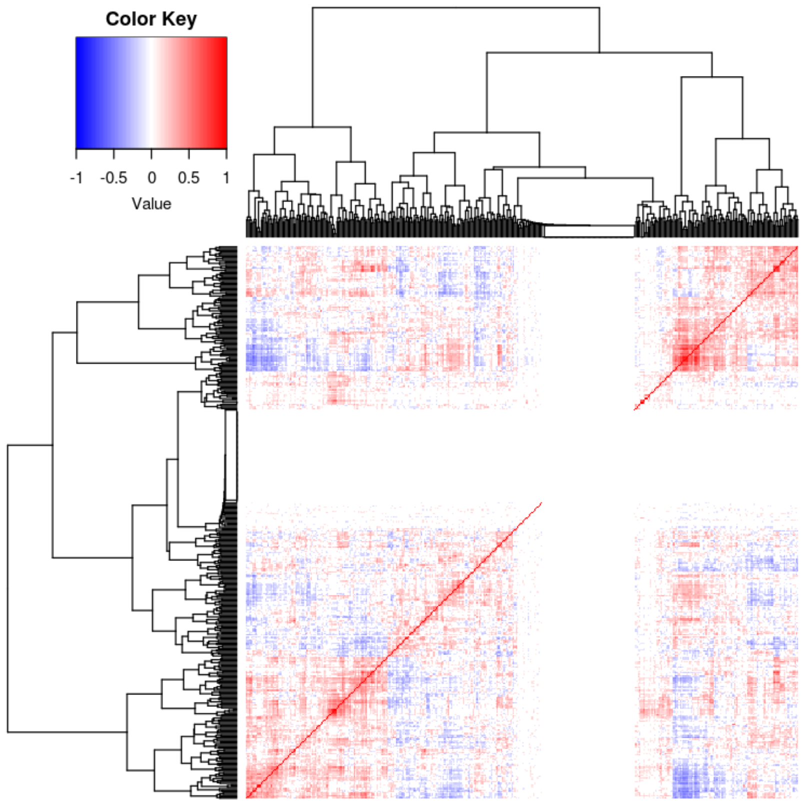

(B) Spearman's correlation coefficient

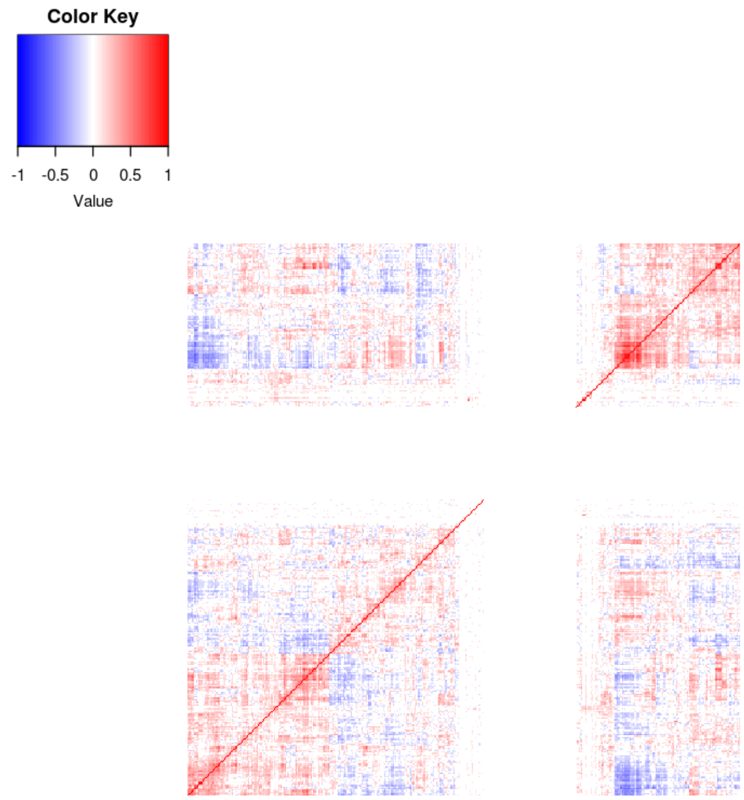

Supplement: Supplementary file 6 — Additional file 6 Heatmaps for correlation matrices by Pearson correlation coefficient (PCC) and Spearman’s correlation coefficient (SCC) in three cancer types. [file 12920_2019_634_MOESM6_ESM.pdf]
